# Neuroimaging and Psychometric Assessment of Mild Cognitive Impairment After Traumatic Brain Injury

**DOI:** 10.3389/fpsyg.2020.01423

**Published:** 2020-07-07

**Authors:** Maria Calvillo, Andrei Irimia

**Affiliations:** ^1^Ethel Percy Andrus Gerontology Center, Leonard Davis School of Gerontology, University of Southern California, Los Angeles, CA, United States; ^2^Denney Research Center, Department of Biomedical Engineering, Viterbi School of Engineering, University of Southern California, Los Angeles, CA, United States

**Keywords:** traumatic brain injury, mild cognitive impairment, neuropsychology, psychometric assessment, neuroimaging, social cognition

## Abstract

Traumatic brain injury (TBI) can be serious partly due to the challenges of assessing and treating its neurocognitive and affective sequelae. The effects of a single TBI may persist for years and can limit patients’ activities due to somatic complaints (headaches, vertigo, sleep disturbances, nausea, light or sound sensitivity), affective sequelae (post-traumatic depressive symptoms, anxiety, irritability, emotional instability) and mild cognitive impairment (MCI, including social cognition disturbances, attention deficits, information processing speed decreases, memory degradation and executive dysfunction). Despite a growing amount of research, study comparison and knowledge synthesis in this field are problematic due to TBI heterogeneity and factors like injury mechanism, age at or time since injury. The relative lack of standardization in neuropsychological assessment strategies for quantifying sequelae adds to these challenges, and the proper administration of neuropsychological testing relative to the relationship between TBI, MCI and neuroimaging has not been reviewed satisfactorily. Social cognition impairments after TBI (e.g., disturbed emotion recognition, theory of mind impairment, altered self-awareness) and their neuroimaging correlates have not been explored thoroughly. This review consolidates recent findings on the cognitive and affective consequences of TBI in relation to neuropsychological testing strategies, to neurobiological and neuroimaging correlates, and to patient age at and assessment time after injury. All cognitive domains recognized by the Diagnostic and Statistical Manual of Mental Disorders (DSM-5) are reviewed, including social cognition, complex attention, learning and memory, executive function, language and perceptual-motor function. Affect and effort are additionally discussed owing to their relationships to cognition and to their potentially confounding effects. Our findings highlight non-negligible cognitive and affective impairments following TBI, their gravity often increasing with injury severity. Future research should study (A) language, executive and perceptual-motor function (whose evolution post-TBI remains under-explored), (B) the effects of age at and time since injury, and (C) cognitive impairment severity as a function of injury severity. Such efforts should aim to develop and standardize batteries for cognitive subdomains—rather than only domains—with high ecological validity. Additionally, they should utilize multivariate techniques like factor analysis and related methods to clarify which cognitive subdomains or components are indeed measured by standardized tests.

## Introduction

Despite inadequate consensus on its definition, traumatic brain injury (TBI) can be described as a clinical condition in which brain function is disrupted due to a traumatic force applied to the head, and whose sequelae can include loss of consciousness (LOC), loss of immediate retrospective and/or prospective memory, mental state changes, and/or focal neurological deficits (American Congress of Rehabilitation Medicine, 1993). By 2013, ∼2.5 million emergency hospital visits in the United States (US) could be attributed to TBI, highlighting this condition as a major public health problem (Taylor et al., 2017). In the United States, TBI is a notable cause of death and disability which predominantly affects vulnerable groups like individuals over the age of 65, children aged 0 to 4, and young adults aged 15 to 24; TBI has higher prevalence in males (Taylor et al., 2017). Patients may experience lower functional independence, greater difficulty in completing their education, more challenges in finding and maintaining gainful employment, and a compromised ability to experience leisure or to maintain meaningful social relationships (Draper and Ponsford, 2008). Depending on injury severity, TBI can be categorized as mild, moderate or severe; 75% to 90% of all cases are typically classified as mild TBI (mTBI), although prevalence is likely higher because many affected individuals do not seek medical care (Prince and Bruhns, 2017).

Recent increases in TBI research breadth, quantity and expenditures by funding agencies highlight the importance of investigating its etiology and neurocognitive sequelae (Prince and Bruhns, 2017). Compared to moderate-to-severe TBI (msTBI), mTBI typically results in fewer and milder adverse consequences, such that its typical victims experience symptom resolution within 3 months (Prince and Bruhns, 2017). However, about 10% to 20% of mTBI patients exhibit long-term post-concussive symptoms (PCSs) marked by connectome disconnection (Irimia et al., 2012, 2014, 2019; Irimia and Van Horn, 2014) as well as by degradation of motor and/or neurocognitive function (Niogi et al., 2008b; Prince and Bruhns, 2017). This percentage may be even higher: according to a comprehensive review of 45 studies, ∼50% of single mTBI patients exhibit long-term mild cognitive impairment (MCI) even after excluding cases which involve litigation or circumstances associated with suspected symptom exaggeration (McInnes et al., 2017).

Common mTBI symptoms include both somatic complaints (headaches, vertigo, sleep problems, nausea, light or sound sensitivity) and affective symptoms (clinical depression, anxiety, irritability and emotional instability) (Prince and Bruhns, 2017). Impaired cognition is typically correlated with poor outcome, even when such impairment is subtle (Niogi et al., 2008b). At all adult ages, mTBI can trigger gradual cognitive decline progressing across the lifespan and frequently affecting attention, processing speed, memory and executive function (EF) (Senathi-Raja et al., 2010; Prince and Bruhns, 2017). Notably, social cognition impairment has been reported up to ∼4 years post-mTBI and up to ∼10 years post-TBI (Ponsford et al., 2013; Theadom et al., 2019) and such impairment is significantly associated with post-concussive quality of life (Jones et al., 2011). Despite these facts, however, impaired social cognition has not been studied comprehensively in TBI samples. For this reason, the first aim of this review is to synthesize recent knowledge on cognitive deficits after mTBI, with emphasis on social cognition impairment.

Adding to the complexity of TBI symptomatology is the fact that heterogeneous brain pathology patterns may arise across a wide variety of injury severities and traumatic mechanisms (e.g., acceleration/deceleration during moving vehicle accidents, direct blunt-force trauma in sports or blast impact during battlefield explosions) (Wu et al., 2016). Notwithstanding injury heterogeneity, the most commonly reported neuroimaging findings after mTBI include cortical thinning in frontal and temporal regions (Draper and Ponsford, 2008; Niogi et al., 2008b; Wu et al., 2016) and traumatic axonal injury (TAI) resulting in additional, widespread white matter (WM) alterations (Irimia and Van Horn, 2013; Sharp et al., 2014). Although studies have attempted to link brain pathology to subsequent cognitive deficits using magnetic resonance imaging (MRI), functional MRI (fMRI) and diffusion MRI (dMRI, including diffusion tensor imaging or DTI), such efforts have not been reviewed sufficiently through the lens of their relevance to the psychometric assessment of mTBI patients. For this reason, the second goal of this review is to discuss such efforts to link neuroimaging to the cognitive assessment of TBI patients. Because this review focuses primarily on psychometrics rather than imaging, the reader is referred to the reviews of (A) Irimia and Van Horn (2015b) for fMRI findings related to post-TBI cognitive deficits, (B) Van Horn et al. (2017) for findings on post-TBI neurometabolic dysfunction, and (C) Irimia et al. (2012, 2014), Goh et al. (2015) for the use of neuroimaging to predict clinical outcome.

Early on, studies used primarily subjective self-reports, and practice shifted only later toward more objective assessment strategies (Draper and Ponsford, 2008). Historically, cognitive tests were utilized primarily to detect brain damage rather than to assess cognitive deficits, which is partly why many tests do not have high ecological validity (Sbordone, 2008). In other words, the assessment of a specific cognitive domain may not capture its deficits adequately because, frequently, measurement techniques are purposely oversimplified to the extent that their results no longer reflect daily-life deficits within that domain. Furthermore, some cognitive tests may not yet have reached the adequate rigor and standardization required before their interpretation is unambiguous (Sbordone, 2008). Coupled with ongoing psychometric challenges related to the adequate formulation of a comprehensive taxonomy of cognition, this can lead to difficulties with assessment utility, interpretation and comparison across studies (Karr et al., 2013). One example involves verbal fluency association tests, which are commonly used by mTBI researchers to assess the ability to produce as many words which start with a given letter as possible within some timeframe. Currently, the extent to which such tests assess executive function (EF) rather than language remains unclear (Whiteside et al., 2016), although some studies classify such tests as assessments of memory (Mueller et al., 2015). Furthermore, cognitive assessments may often detect deficits within more than one cognitive domain, such that their statistical sensitivity can differ based on which cognitive domains and/or deficits are being assessed (Karr et al., 2013). Thus, methodological and interpretative challenges may arise when researchers use the same test to assess different cognitive domains, or even distinct subdomains within a specific domain. Conversely, difficulties may also ensue when researchers utilize different tests to assess the same cognitive domain. This may be because neuropsychological tests vary greatly in their suitability to measure mTBI-related cognitive deficits even within a single cognitive domain (Draper and Ponsford, 2008; Karr et al., 2013). Alternatively, this could be due to the multifaceted nature of cognition, as the tests in question may, in fact, quantify two different subdomains or abilities within the same cognitive domain (Sachdev et al., 2014). To improve cross-study comparison and to facilitate rigorous, comprehensive meta-analysis of cognitive mTBI research, such inaccuracies must be identified and resolved. In light of the above, the ability to detect cognitive impairments after mTBI is partly reliant upon how such impairments are assessed (Prince and Bruhns, 2017). Because this important methodological aspect has not been investigated sufficiently, the third aim of this review is to summarize and evaluate the use of cognitive tests after mTBI and to provide recommendations on their prudent utilization.

Although mTBI-related cognitive deficits are routinely examined in research studies, the accurate comparison of cognitive assessments *across* studies is an arduous task due to the complexities of cognitive testing and owing to mTBI heterogeneity. For this reason, we here review recent research on cognitive dysfunction after mTBI from the standpoints of psychometric assessment strategies, neural correlates, and important variables like age at injury and the assessment time post-injury. Due to imperfect consensus on the taxonomy of cognitive (sub)domains, this review relies upon the categorizations proposed by the Diagnostic and Statistical Manual of Mental Disorders 5 (DSM-5) to formulate guidelines (Sachdev et al., 2014). Each domain and subdomain is reviewed with a focus on aging adults; pediatric mTBI assessment is beyond our scope. Although we focus on mTBI, important findings on msTBI are also discussed whenever mTBI data are unavailable, or when msTBI findings are particularly illuminating.

## Depression, Anxiety, and Effort

Individuals with a history of mTBI are at relatively high risk for post-concussive depressive symptoms (PCDSs), for anxiety and for irritability marked by lack of patience, aggression and emotional instability (Arciniegas and Wortzel, 2014; Prince and Bruhns, 2017). Emerging evidence suggests that disturbance of serotonin production in the gastrointestinal tract after TBI may be related to such symptoms (Irimia and Bradshaw, 2003, 2005a,b; Irimia et al., 2015). Whereas emotional and behavioral disturbances are common early after injury, such manifestations are thought to resolve mostly within weeks (Arciniegas and Wortzel, 2014). By contrast, these symptoms can become more limiting after msTBI, as they are often more intense and can persist longer (Arciniegas and Wortzel, 2014). Post-concussive anxiety and PCDSs are the most common emotional disturbances following mTBI, and their magnitude often predicts that of other symptomatology in addition to functional outcome and recovery (Mooney and Speed, 2001; Meares et al., 2008; Levin and Diaz-Arrastia, 2015; Zahniser et al., 2018). Above and beyond affect-related symptoms, studying the poor ability, desire or motivation to invest effort into cognitive tasks after injury is also fundamental because inadequate effort—which has been reported in many TBI samples—can significantly confound psychometric measures (Stulemeijer et al., 2007). Thus, before reviewing findings pertaining to cognition, we discuss affect and effort.

### PCDSs

PCDSs have been noted both acutely (for both complicated and uncomplicated mTBI) and up to ∼1 year after mTBI (regardless of complications), with improvements over time (Panayiotou et al., 2010; Ponsford et al., 2011; Lucas et al., 2016). PCDS prevalence after injury has been reported to range from ∼10% to ∼77% across all injury severities (∼14% to ∼53% for post-traumatic major depressive disorder) and from ∼15% to ∼35% after mTBI in particular. Most PCDSs occur in the 1st year after injury, although risk can remain elevated for many years (Rapoport et al., 2003; Silver et al., 2009; Bombardier et al., 2010). Such risk can be influenced by (A) pre-existing conditions (e.g., substance abuse, mental/personality disorders), (B) injury mechanism (e.g., sports injury, vehicle accidents), (C) TBI anatomic profile (e.g., frontotemporal, fronto-occipital), (D) neurochemical imbalances, (e.g., disruption of serotonergic neurotransmission, excessive glutamatergic signaling), (E) injury-related comorbidities, including other PCSs and (F) socioeconomic factors (e.g., level of social support during recovery) (Silver et al., 2009). PCDSs are associated with increases in the number and severities of other self-reported symptoms in TBI patients, surpassing those reported by non-concussed, clinically depressed outpatient groups (Silver et al., 2009; Lange et al., 2011). Partly for this reason, PCDSs can promote a vicious cycle in which increased aggression, cognitive dysfunction and suicidal thoughts lead to a perception of lower life quality, which in turn exacerbates PCDSs. Typically, mTBI patients with PCDSs are significantly older than those who lack them; this is consistent with the hypothesis that advanced age is a PCDS risk factor (Levin et al., 2005; Rao et al., 2010). PCDSs may be responsible for—or contribute to—cognitive dysfunction after TBI. For example, Himanen et al. (2009) compared the cognitive performances of TBI participants with or without a clinical depression diagnosis and suggested that, whereas complex attention deficits are frequently linked to TBI, depression may be responsible for some deficits of psychomotor speed and sustained attention.

Neuroimaging correlates of PCDSs often involve frontal brain regions; for example, a computed tomography (CT) lesion study found that frontal subdural lesions in mTBI are significantly associated with chronic PCDSs (Rao et al., 2010). Upon analyzing the resting-state (rs) fMRI signals of sub-acute mTBI participants, Zhou et al. (2012) found that stronger functional correlations (FCs) between regions within medial prefrontal cortex (mPFC) were significantly and inversely associated with self-reported PCDS ratings. dMRI studies have found that mTBI-related WM damage in medial and subcortical frontal regions—including the corpus callosum, corona radiata, superior longitudinal fasciculus, anterior cingulum, corticostriatal and thalamo-frontal projections—can be associated with PCDSs, including lack of interest, lethargy, low drive and lack of initiative (Matthews et al., 2011; Zappalà et al., 2012).

### Anxiety

Post-traumatic anxiety has been documented acutely, up to ∼6 months after uncomplicated mTBI and up to ∼5.5 years after TBI of greater severity (Dischinger et al., 2009; Meares et al., 2011; Van Der Naalt et al., 2017). As in the case of PCDSs, anxiety symptoms are relatively frequent in the 1st year after TBI (∼19% to ∼70% prevalence) and typically include generalized anxiety disorder (GAD) and post-traumatic stress disorder (PTSD), both of which are usually characterized by poor clinical resolution (Whelan-Goodinson et al., 2009; Gould et al., 2011). Nevertheless, anxiety symptoms may also improve over time; for example, Ponsford et al. (2011) found that mTBI and trauma control groups exhibited substantial symptom reductions within ∼3 months post-injury. Like in the general population, anxiety symptoms are often comorbid with those of depression, which may compound each other’s negative effects upon recovery (Gould et al., 2011). In a group of msTBI patients, prior history of anxiety disorders increased post-traumatic anxiety risk by a factor of 9.47, with every additional post-injury month increasing this risk by 11% (Gould et al., 2011). One year post-mTBI, greater anxiety was found to be significantly and inversely correlated with WM volume changes in the isthmus of the left cingulate gyrus, supporting previous associations between frontal WM loss and anxiety after TBI (Zhou et al., 2013). However, no correlation has been found between anxiety after mTBI and fMRI measures (Shumskaya et al., 2012; Tang et al., 2012; Zhan et al., 2015).

### Effort

In the context of neuropsychological testing, effort can be defined as the amount of energy invested (sub)consciously into completing neuropsychological assessment tasks. Some patients with a history of mTBI have been found to exaggerate their reports of injury severity and of subsequent cognitive impairment compared to patients with a history of msTBI (Prince and Bruhns, 2017). In fact, effort may affect mTBI patients’ neurocognitive test performances more than injury severity (West et al., 2011). Patients who perform poorly on symptom validity tests (which can be suggestive of poor effort) often report relatively more serious injury-related sequelae and perform worse on cognitive tests across all domains compared to good-effort performers (Stulemeijer et al., 2007; Prince and Bruhns, 2017). Litigation status has not been associated with poor effort after mTBI, although patients in litigation frequently report worse or diminishing cognitive function compared to non-litigants (Belanger et al., 2005; Stulemeijer et al., 2007).

Although the cause of poor effort during post-mTBI cognitive assessments remains unclear, Prince and Bruhns (2017) have summarized evidence indicating that this phenomenon may be partially due to a dysfunctional feedback loop in which affective, somatic and cognitive symptoms interact to prolong the subjective perception of symptom severity, even when the primary cause of the symptoms has been resolved. For example, in the study of Silverberg et al. (2017), mTBI patients fearing mental exertion due to the expectation of a subsequent headache scored worse on memory performance tests than those without such a fear. Consistent with the dysfunctional feedback loop hypothesis, Van Der Horn et al. (2015) suggested that, because mPFC is involved prominently in emotional regulation, default mode network (DMN) hyperactivity and associated mPFC dysfunction are very likely linked to PCS persistence after mTBI. Supporting this notion, the positron emission tomography (PET) study of Spadoni et al. (2015) found that chronic mTBI participants who had invested relatively little effort into their psychometric assessment tasks exhibited significantly lower glucose metabolism in ventromedial PFC compared to participants investing an adequate amount of effort. Furthermore, affective factors can be better PCS predictors than neuropsychological test measures; thus, such symptoms are likely more intimately linked to mTBI-related psychological disturbances rather than to genuine neurobiological changes, as detailed by Clarke et al. (2012). However, in these authors’ study, neuropsychological test performance predicted cognitive complaint severity in the mTBI—but not in the control—group; this suggests the existence of genuine—albeit subtle—PCS-related cognitive deficits which do not have a strictly psychological substrate. A second explanation involves the “good old days” bias, according to which patients are more likely to perceive their pre-injury state as better than it truly was. Supporting this theory, Lange et al. (2010) found that chronic mTBI participants retrospectively report a significantly lower intensity and number of pre-injury symptoms compared to healthy controls (HCs). This effect is present in both litigating and non-litigating mTBI participants and is not affected by age at injury or by educational attainment. Although additional research on post-traumatic malingering is needed, most evidence reviewed here suggests that it is unlikely to be premeditated. In conclusion, PCDSs, anxiety and poor effort are important factors to consider when assessing cognitive function in mTBI, as each of these (A) has a relatively high post-concussive prevalence and (B) can have considerable effects upon psychometric performance.

## Social Cognition

Post-concussive difficulties in communication and in the maintenance of social relationships have been documented both acutely and up to ∼10 years post-injury, with the prevalence of such manifestations increasing over time (Ponsford et al., 2013). In one study, ∼10 years post-injury, ∼30% of msTBI patients reported problems with personal relationships, including friend loss and isolation (Knox and Douglas, 2009; Ponsford et al., 2013). Notably, social dysfunction symptoms are typically far more prevalent and severe after msTBI rather than mTBI, which may be why many researchers either do not stratify samples based on severity or instead choose not to study mTBI participants (Arciniegas and Wortzel, 2014). Improving our understanding of social dysfunction after TBI is important because social difficulties can impact post-concussive quality of life significantly (Jones et al., 2011; Spikman et al., 2013; Proctor and Best, 2019). The positive impact of research-informed strategies for the education and social support of TBI patients is highlighted by the finding that more severe head injuries can in fact lead to significantly better functional outcomes if patients report healthy social relationships and a strong sense of personal identity (Jones et al., 2011). Although many factors can contribute to post-concussive social dysfunction (i.e., social challenges related to emotion recognition, perspective-taking or altered self-awareness), its mechanisms remain unclear (Milders et al., 2008; Babbage et al., 2011; Arciniegas and Wortzel, 2014). However, they seem to be independent of—and not likely caused by—other cognitive deficits (Spikman et al., 2011).

### Emotion Recognition

Deficits of facial emotion recognition are frequent after TBI and have been associated with poorer social functioning outcomes (Knox and Douglas, 2009; Babbage et al., 2011). Such deficits have been recorded both acutely and up to ∼5 years post-injury, mostly in msTBI samples (Babbage et al., 2011; Spikman et al., 2011). One meta-analysis found that 13% to 39% of individuals with msTBI have significant deficits of emotion recognition, performing approximately over one standard deviation below HC participants’ mean scores on measures of facial affect recognition (Babbage et al., 2011). Furthermore, emotion recognition impairments after mTBI have been recorded using several stimulus types (i.e., recognition, matching, labeling, discrimination) and appear to be persistent (Ietswaart et al., 2008; Knox and Douglas, 2009; Babbage et al., 2011). Like HCs, individuals with TBI often exhibit greater impairment, both acutely and chronically, in the recognition of negative—rather than positive—emotions, which reflects a normal response to task difficulty (Ietswaart et al., 2008). Although emotion recognition may be impacted by mTBI, there are few studies to confirm this finding (Babbage et al., 2011) and measurable effects might be confounded by PCDSs (Bourke et al., 2010). The mechanism whereby impaired emotion recognition results in poorer post-TBI social functioning is unclear; nevertheless, the former has been suggested to lead to poorer comprehension of oral communication, resulting in inability to evaluate the appropriateness of one’s social behavior and in unsuitable social responses (Knox and Douglas, 2009). One potential cause for emotion recognition challenges after TBI is alexithymia, i.e., the inability to identify and describe emotions in oneself and/or others (Williams and Wood, 2010). The high post-traumatic prevalence and severity of this condition have been linked to relatively lower emotional empathy in chronic TBI, leading to further social challenges (Williams and Wood, 2010).

Poor emotion recognition after chronic msTBI is significantly associated with damage to orbitofrontal cortex (Spikman et al., 2011) which is expected given this region’s involvement in social cognition. In a TBI sample of mixed severity imaged ∼9 years post-injury, Neumann et al. (2016) uncovered a significant inverse relationship between emotion recognition impairment and task-related fMRI activation of the right fusiform gyrus, which is involved in facial recognition and visual perception. Poor emotion recognition about 10 years after msTBI is also significantly related to reduced WM integrity in the inferior longitudinal and fronto-occipital fasciculi, and with reduced gray matter (GM) volume in the lingual and parahippocampal gyri (Genova et al., 2015). These findings are not surprising given that these structures are involved in high-level social interaction, memory retrieval and visual (particularly facial) processing (Natu and O’Toole, 2011; Sarubbo et al., 2013; Catani and Bambini, 2014).

### Theory of Mind

Theory of mind (ToM) deficits after TBI have been recorded both acutely and up to ∼3 years post-injury (Milders et al., 2008; Spikman et al., 2011). Such impairments have been demonstrated using both verbal and non-verbal measures, and appear to be persistent throughout the 1st year post-injury (Milders et al., 2008). ToM impairments after TBI include difficulties with (A) understanding and explaining the feelings and intentions of others, (B) correctly identifying non-faux pas scenarios (while over-reporting faux-pas scenarios due to uncertainty), (C) understanding indirect speech (including humor and sarcasm, regardless of type) and (D) inhibiting self-referential thoughts when considering another person’s perspective (Channon et al., 2005; Milders et al., 2006; Martín-Rodríguez and León-Carrión, 2010; McDonald et al., 2014). Interestingly, in a group of mixed-severity TBI patients, Milders et al. (2006) found that injury severity did not affect ToM performance or its course over time, although further evidence is needed. Because ToM tasks often require adequate EF and language abilities, ToM-related impairments may stem from TBI-related executive and/or speech dysfunction, and particularly from deficits of cognitive flexibility, inhibition, phonemic fluency or working memory, which are all significantly and positively correlated with ToM impairments in both acute and chronic TBI (Henry et al., 2006; Milders et al., 2008; McDonald et al., 2014; Honan et al., 2015). Nevertheless, it is likely that some post-TBI ToM deficits are independent of other cognitive impairments, given TBI patients’ poor performance on non-verbal ToM tests and on ToM tests with low EF demands (Havet-Thomassin et al., 2006; Milders et al., 2008; Geraci et al., 2010; Martín-Rodríguez and León-Carrión, 2010; McDonald et al., 2014; Bosco et al., 2017). For example, McDonald et al. (2014) studied chronic TBI patients’ performance on a ToM test requiring varying levels of both EF (i.e., low EF, high inhibition, high flexibility) and ToM engagement (low-ToM engagement, high-ToM engagement). The authors found that variability of participants’ performance on the low-ToM engagement task made a unique contribution to the variance of their performance on the high-ToM engagement task for conditions requiring low EF and high flexibility (but not high inhibition). The conclusion of the study was that EF and ToM may contribute independently to ToM performance after TBI in some cases, such as when high inhibition is needed.

Frontal lobe damage has been repeatedly tied to poor post-TBI performance on faux pas tests, which are commonly used to assess ToM (Martín-Rodríguez and León-Carrión, 2010). While mixed-severity chronic TBI groups with (A) ventromedial and (B) dorsolateral PFC damage performed equally poorly on the Reading the Mind in the Eyes (RME) Test (commonly used to assess social perception and ToM), only participants with localized damage to ventromedial PFC performed poorly on the faux pas test (Geraci et al., 2010). Additionally, poor performance on the RME test in chronic penetrating TBI was significantly associated with damage to the left inferior frontal gyrus (Dal Monte et al., 2014). Thus, although further research is needed to ascertain the neuroimaging correlates of ToM impairment after TBI, it comes as no surprise that frontal lobe damage is critically involved in such deficits. More studies are required to characterize the extent and neural correlates of ToM dysfunction in mTBI patients.

### Self-Awareness

As in the case of ToM and emotion recognition, self-awareness (SA) deficits have been found mostly after msTBI rather than after mTBI (Bar-Haim et al., 2009; Arciniegas and Wortzel, 2014; Gaines et al., 2016). SA deficit prevalence after mixed-severity TBI has been estimated to range from 45 to 97%, with higher prevalence being weakly linked to greater injury severity (Sherer et al., 1998; Bach and David, 2006). These deficits have been recorded after mixed-severity TBI acutely up to ∼1 year after injury (including after both complicated and uncomplicated mTBI) and up to ∼5 years after msTBI only (Sherer et al., 2003; Hart et al., 2009; Kelley et al., 2014). Longitudinal studies have consistently reported SA improvements between the acute and chronic stages of TBI, particularly after severe TBI (Hart et al., 2009; Ponsford et al., 2013). In a sample of mixed-severity acute TBI patients, older age and better functional independence were both significantly associated with improved SA ratings (Sherer et al., 2003). Notably, higher SA was found to be significantly correlated with increased self-esteem, with lower depression ratings and with improved employability, thus illustrating the clinical importance of SA recovery (Sherer et al., 1998, 2003; Carroll and Coetzer, 2011).

SA deficits can differ across injury severities (Arciniegas and Wortzel, 2014). In one study, for example, patients with msTBI report irritability levels closer to those of HCs, whereas their caregivers reported that patients exhibited considerably higher levels (Arciniegas and Wortzel, 2014). On the other hand, mTBI participants’ self-reported irritability levels were similar to those reported by the msTBI participants’ caregivers (Arciniegas and Wortzel, 2014). Thus, whereas some mTBI patients’ altered SA may lead them to exaggerate the magnitude of their symptoms, the SA of many individuals with msTBI may be altered to underestimate symptoms (Sherer et al., 2003; Arciniegas and Wortzel, 2014). SA impairments may also differ based upon the nature of the specific deficits involved and upon the phrasing of questions asked during assessment (Sherer et al., 2003). For example, greater TBI-related SA challenges are usually noted in reports of cognitive and behavioral impairments (rather than in reports of physical deficits) and, furthermore, in response to general questions rather than to specific ones (Sherer et al., 2003). It has been suggested that SA deficits can be connected to inadequate ToM after TBI. Specifically, Bivona et al. (2014) found that poor SA in severe chronic TBI patients is linked to worse performance on the Faux Pas Test and on the First Order False Belief Test, compared to HCs and to TBIs with better SA. A significant positive association between SA and emotion recognition in chronic msTBI has also been proposed (Spikman et al., 2013); thus, deficits in abilities which are integral to optimal social functioning may be substantially comorbid after injury.

Using fMRI, Schmitz et al. (2006) found that participants with chronic TBIs of mixed severities and with poor SA exhibited greater bilateral activation of anterior cingulate cortex (ACC), of the precuneus and of the right temporal pole during a self-appraisal task. Better SA was linked to greater task-related activation of the right anterior dorsal PFC. These findings are not surprising given previous associations between these structures, on the one hand, and interoceptive/emotional awareness, self-reflection and consciousness on the other hand (Critchley et al., 2004; Schmitz et al., 2004; Cavanna, 2007; Legrand and Ruby, 2009). Because only one neuroimaging study on the correlates of SA with TBI could be located, further research on this topic should be undertaken.

## Complex Attention

Deficits of complex attention are among the most commonly reported consequences of TBI (McInnes et al., 2017). According to the DSM-5, complex attention includes the subdomains of sustained, divided and selective attention in addition to processing speed, which is frequently assessed as a stand-alone ability (Sachdev et al., 2014). Deficits within the overall domain of attention have been noted in both the acute and chronic stages of mTBI, although conflicting results have been reported. Specifically, some research studies and meta-analyses found significant attention deficits as late as ∼6 years post-mTBI (∼10 years after msTBI) whereas others reported no such deficits ∼3 months post-injury (Draper and Ponsford, 2008; Konrad et al., 2011; Rohling et al., 2011; McInnes et al., 2017). These conflicting results may be partly explained by the fact that most studies reviewed here fail to distinguish between complicated and uncomplicated mTBI. This often leads to study results and conclusions being based on samples with both complicated and uncomplicated mTBI. For example, the study which concluded that post-concussive cognitive deficits dissipate within 3 months post-injury was one of the few studies which included only patients with uncomplicated mTBI; this suggests the possibility of better cognitive recovery for uncomplicated—as opposed to complicated—mTBI (Rohling et al., 2011). Furthermore, although it is frequently assumed that cognitive deficits—including attentional dysfunction—diminish with time (McInnes et al., 2017), this phenomenon is insufficiently understood and its presentation may depend on factors like age at injury (Prince and Bruhns, 2017). One study by Senathi-Raja et al. (2010) which did not account for injury severities and in which participants were tested ∼10 to ∼12 years post-injury suggested that, in young TBI participants (16–34 years), longer time since injury is linked to better attention performance. In middle-aged TBI participants (35–54 years), no relationship was found between time since injury and attention; in older TBI participants (55 years or older), longer time since injury was linked to poorer attention performance. Thus, recovery from attention deficits likely depends on both age at injury and on the time after injury when attention is assessed, and future research should account for these variables (Halgren et al., 2011). In their study, Senathi-Raja et al. also found that, relative to age-matched HCs, older individuals who had suffered a TBI of any severity exhibited a significantly wider attention performance gap compared to that of persons who had been injured at a younger age. More research should be undertaken to clarify how age at injury affects attention after mTBI.

In a group of middle-aged mTBI patients imaged ∼5 years after injury using structural MRI, poorer performance on attention tasks was found to be associated with reductions in both WM—in cingulate, parietal and occipital cortices—and GM, in temporal cortex (Little et al., 2014). One fMRI study of chronic mTBI reported increased ACC activation and decreased PFC activation during attention tasks (Dean et al., 2015). Upon using fMRI to measure rs-FCs after msTBI, Shumskaya et al. (2017) found that patients exhibited poorer attention and stronger FCs involving the sensorimotor network compared to HCs. A significant positive correlation between attention and rs-FCs in this network was found in the TBI group, whereas the HC group exhibited a significant negative FC between these measures. Using dMRI, one study which did not stratify participants based on injury severity found that, compared to HCs, TBI participants exhibited a significant negative correlation between the number of low-integrity WM fasciculi and overall attention performance (Kraus et al., 2007). Future studies should aim to examine how structural brain circuitry differs in mTBI patients as a function of their performance on attention tasks.

### Sustained Attention

mTBI patients can exhibit deficits of sustained attention both acutely and up to ∼2 years post-injury (Chan, 2005; Kwok et al., 2008; Pontifex et al., 2012; Azouvi et al., 2017). In these studies, no distinction was made between complicated and uncomplicated mTBI, with the exception of the study by Kwok et al. Measures of TBI severity—like the Glasgow Coma Score (GCS), LOC duration and the extent of post-traumatic amnesia (PTA)—have been found to be significantly correlated with poorer sustained attention (Chan, 2005). Notably, whereas other types of attention improve over 3 months following mTBI, sustained attention remains relatively poor, as shown by a study of complicated mTBI (Kwok et al., 2008). MRI findings suggest that, ∼1 month post-injury, mTBI patients’ deficits of sustained attention are associated with cortical volume loss in the right ventral ACC (Zhou et al., 2013). Upon combining dMRI with fMRI, Bonnelle et al. (2011) found that sustained attention impairments observed ∼2 years post-injury were associated with increased task-related DMN activation involving the precuneus and posterior cingulate cortex (PCC), which is suggestive of inefficient information processing during sustained attention. DMN disconnection extent—particularly involving the precuneus—was related to TBI participants’ performance, with broader disconnection linked to poorer sustained attention.

### Divided Attention

Divided attention impairment after mTBI has been recorded up to ∼4 years post-injury, typically with improvement over time (Mangels et al., 2002; Kwok et al., 2008; Paré et al., 2009). The only study which distinguished between complicated and uncomplicated mTBI was that of Kwok et al. Older studies are more likely to report conflicting results as to whether or not divided attention is impaired by mTBI, owing to confounds like (A) different cognitive loads imposed by different tests, (B) failure to account for time since injury and (C) failure to control for processing speed deficits (Paré et al., 2009). For example, evidence for TBI-related deficits of divided attention has most frequently been found using relatively complex tasks requiring high cognitive loads, and when assessing cognitive control rather than speed (Beaulieu-Bonneau et al., 2017). Divided attention deficits may underlie impairments of memory consolidation and recognition: in one study, both mild and severe TBI patients performed poorly on tests of divided attention and their performance was associated with their episodic memory performance, although this relationship was only significant for the severe TBI group (Mangels et al., 2002). By comparing mTBI patients’ acute MRI scans to those obtained ∼1 year post-injury, Dall’acqua et al. (2017b) found that participants with relatively poor clinical outcome exhibited a significant relationship between greater PFC thickness and poorer divided attention (cortical thickening possibly being due to neuroinflammation). In an fMRI study of mTBI patients imaged both acutely and ∼1 year post-injury (Dall’acqua et al., 2017a), researchers found that, compared to HCs, the mTBI group exhibited task-related DMN hypoactivity (bilaterally: ACC, PCC, precuneus, Heschl’s gyrus, superior temporal gyrus and temporal pole; right hemisphere: parahippocampal gyrus, amygdala, and supplementary motor area); rs-FC strength was significantly and negatively correlated with performance on a divided attention task.

### Selective Attention

After mTBI, selective attention can be impaired both acutely and up to 7–8 months post-injury, with reported improvements over time (Ziino and Ponsford, 2006; Dall’acqua et al., 2017b). These studies did not distinguish between complicated and uncomplicated mTBI. There are contradictory findings on mTBI effects upon selective attention, possibly due to differing neuropsychological testing methodologies (Beaulieu-Bonneau et al., 2017). For example, upon examining selective attention ∼8 months post-injury, Ziino and Ponsford (2006) found impairments of selective attention during relatively complex tasks even after controlling for PCDSs and anxiety. By contrast, participants’ impairment on relatively simpler selective attention tasks was explained by comorbid depression, anxiety and fatigue. This illustrates the importance of accounting for affective factors when quantifying attention performance after mTBI. Selective attention performance after mTBI may be influenced by additional factors; for example, it is uncertain whether LOC after mTBI is related to changes in selective attention. Whereas some studies indicate that mTBI patients with acute LOC perform worse on tests of selective attention, others do not (Carroll et al., 2014; De Freitas et al., 2019). One meta-analysis suggests that TBI-related deficits in selective attention on certain tasks, such as on the widely used Stroop interference task, may be largely due to the downstream effect of slower processing speed, which is frequently reported after TBI (Ben-David et al., 2011). However, it is still unclear whether this downstream effect occurs during other common selective attention tasks.

An MRI study of mTBI participants with good outcomes ∼1 year post-injury found subtle cortical PFC thickening—which may be due to chronic neuroinflammation—linked to improvements in selective attention (Dall’acqua et al., 2017a). In an fMRI study, Mayer et al. (2012) suggests that mTBI patients’ DMNs are intimately involved in modulating selective attention performance; for example, unlike HCs, mTBI participants failed to deactivate their DMNs in response to selective attention tasks at high cognitive load. Also unlike HCs, mTBI participants failed to exhibit typical attention-related modulations in their neuronal responses during a selective attention task. In a group of mTBI adults imaged approximately ∼1 month after injury, Smits et al. (2009) found increased blood oxygenation level-dependent (BOLD) signals in the ventrolateral PFC, posterior parietal lobe and cingulate gyrus during a selective attention task; in this sample, relative BOLD signal strength and PCS severity were correlated. More than a month after mTBI, Niogi et al. (2008a) found that selective attention was significantly correlated with dMRI-measured WM integrity in the left anterior corona radiata, and that the integrity of WM innervating these regions was significantly reduced in mTBI participants compared to HCs.

### Processing Speed

Impaired processing speed is perhaps the most frequently reported cognitive deficit after mTBI. It has been reported acutely, up to ∼6 years after mTBI and up to ∼10 years after TBIs of mixed severity (Mathias et al., 2004; Draper and Ponsford, 2008; Konrad et al., 2011; Dean and Sterr, 2013). These studies did not distinguish between complicated and uncomplicated mTBI. The phenomenon is strongly associated with self-reported fatigue, which is also very common after TBI (Johansson et al., 2009; Ponsford et al., 2013). Johansson et al. (2009) found that the severity of TBI participants’ reported fatigue was not related to injury severity, to their age at injury or to the time after injury when assessments were made, and that the latter factor did not have any significant effect on processing speed. By contrast, in a study of mixed-severity TBIs, Senathi-Raja et al. (2010) concluded that, for young participants, longer time since injury was associated with improved processing speed. For middle-aged participants, there was no relationship between the two variables, whereas for older participants remoter injuries were associated with slower processing speed. These apparent differences in results can be reconciled if one takes into account that the sample of Johansson et al. (2009) consisted entirely of middle-aged participants based on the age range criteria of Senathi-Raja et al. In addition, the latter authors found that older age at injury was associated with slower processing speed, although further research is needed for confirmation. Thus, although some evidence suggests that time since and age at injury can be strong modulators of processing speed improvements, further research is needed to clarify their relationship to processing speed after mTBI. Because processing speed influences nearly all cognitive responses to task stimuli and is assessed by a wide variety of neuropsychological tests, poor processing speed often has a downstream effect upon many other cognitive metrics (Beaulieu-Bonneau et al., 2017). Some researchers even assert that processing speed deficits may underlie nearly all observed TBI-related attention deficits, although others maintain that attention deficits are present after mTBI regardless of processing speed impairments (Beaulieu-Bonneau et al., 2017).

Using MRI, Cole et al. (2018) found that processing speed impairment was significantly and positively correlated with the difference between chronological and biological brain age, thus relating greater atrophy to slower processing speed. Using fMRI, Palacios et al. (2017) found that, after acute mTBI, both processing speed and overall attention were significantly and positively correlated with rs-FC in the DMN, in the salience network and in the dorsal attention network. The association of processing speed with such widespread neuroimaging alterations is not surprising given the importance of this fundamental parameter to most other cognitive processes. A dMRI study by Niogi et al. (2008b) found that processing speed—as measured by reaction time—was positively correlated with WM damage in the anterior corona radiata (41% of patients), uncinate fasciculus (29%), genu of the corpus callosum (21%), inferior longitudinal fasciculus (21%), and cingulum bundle (18%). Thus, although further research is needed, it appears that fronto-temporal WM connections may play an important role in the decline and recovery of processing speed performance after mTBI.

## Learning and Memory

Along with attention impairments, deficits of learning and memory (L&M) are among the most commonly reported symptoms of TBI (McInnes et al., 2017). According to the DSM-5, the L&M cognitive domain includes both declarative L&M (i.e., free/cued recall, recognition memory, and semantic/autobiographical long-term memory) and non-declarative L&M, i.e., implicit learning (Sachdev et al., 2014). Overall L&M deficits have been recorded acutely after both complicated and uncomplicated mTBI, including up to ∼6 years following mTBI, and up to ∼10 years after TBIs of mixed severity in studies where the distinction between complicated and uncomplicated mTBI was not made (Draper and Ponsford, 2008; Stulemeijer et al., 2010; Konrad et al., 2011; McInnes et al., 2017). Acute mTBI patients’ performance on L&M psychometric assessments is typically negatively correlated with injury severity (Stulemeijer et al., 2010). Nevertheless, L&M have not always been reported to worsen after injury, potentially due to biological factors (e.g., age at injury, time since injury) and/or methodological confounds across studies (Konrad et al., 2011; Rohling et al., 2011). One study examining TBIs of all severities found that older age at injury was associated with poorer L&M ∼30 years post-injury (Himanen et al., 2006). Interestingly, some mTBI-related deficits of overall memory may be due to the downstream effects of impaired EF or attention upon information encoding and retrieval (Prince and Bruhns, 2017). For example, Mangels et al. (2002) found that chronic mTBI patients exhibited memory recall impairments only when their memory encoding involved divided rather than focused attention (the latter being less demanding). Importantly, the manifestations of TBI-related L&M deficits are typically different from those observed in amnestic disorders like Alzheimer’s disease (AD) (Rabinowitz and Levin, 2014). Whereas amnestic disorders are prominently associated with memory storage deficits, TBI more often features dysfunctional memory encoding mechanisms, whose deficits impact memory retrieval (Rabinowitz and Levin, 2014). For example, individuals with TBI may recall information improperly or may associate unrelated pieces of information together.

The MRI study of Little et al. (2014) linked poor overall memory performance ∼5 years post-injury to tissue volume reductions in the parahippocampal gyri, anterior temporal lobes and internal capsule. Using fMRI, Ge et al. (2009) found that, ∼2 years post-injury, the thalami of mTBI participants exhibited task-related cerebral blood flow (CBF) which was significantly weaker than in HCs, and that CBF decreases were significantly and negatively correlated with volunteers’ overall memory performance. Utilizing dMRI, Niogi et al. (2008a) studied mTBI participants about ∼1.3 years post-injury and found that their overall memory performance was significantly and positively correlated with the integrity of the uncinate fasciculus. Finally, ∼2 years post-injury, the overall memory performances of individuals with TBIs of mixed severity as well as of HC volunteers were found to be associated with WM damage in the fornices (Kinnunen et al., 2010). Although the specificity of these neuroimaging correlates is constrained by the neuropsychological tests utilized to assess overall memory in each study, the involvement of the thalamus, of the anterior and medial temporal lobes and of their connections is not surprising, given the established association between these neuroanatomic structures and memory processing (Simmons and Martin, 2009; Burgmans et al., 2011; De Zubicaray et al., 2011; Leszczynski and Staudigl, 2016).

### Free and Cued Recall

Free and cued recall are concepts used by neuropsychologists to assess (non-) verbal memory, episodic (autobiographical) memory, semantic memory, etc. Due to the wide usage of these paradigms in memory research, one can draw from many of the findings on overall L&M performance after mTBI discussed above to understand free and cued recall. Among mTBI patients, impairments of free and cued episodic memory recall have been found both acutely (for both complicated and uncomplicated mTBI) and up to ∼6 years post-injury (where no distinction between complicated and uncompliated mTBI was made) (Konrad et al., 2011; McCauley et al., 2013). However, cued recall is typically less impaired than free recall; this appears to support the hypothesis that mTBI is not associated with a true dysfunction of memory storage, but rather with dysfunctional encoding mechanisms which impact retrieval (Konrad et al., 2011). The performance of mTBI patients on free and cued recall tasks has been found to improve after a period ranging from 1 month to 1 year post-injury (Dikmen et al., 2016).

### Recognition Memory

No impairments in recognition memory have been reported either after acute TBI or up to ∼6 years post injury in studies which did not distinguish between complicated and uncomplicated mTBI (Mathias et al., 2004; Konrad et al., 2011). Nolin (2006) confirmed mTBI-related impairments in both free and cued recall, but not in recognition memory; once again, these findings support the hypothesis that mTBI can lead to deficits of memory encoding and retrieval, rather than to genuine deficits of memory storage. Notably, some studies use the terms *cued recall* and *recognition* interchangeably, leading to difficulties in identifying research findings on these similar—albeit non-synonymous—concepts (Nolin, 2006; Konrad et al., 2011). Thus, when examining the TBI literature on recognition memory and on cued recall, great caution should be exerted in ascertaining differences in nomenclature across studies. Further research is required to ascertain whether mTBI affects recognition memory.

### Semantic and Autobiographical (Episodic) Memory

Impairments of episodic and semantic memory after mTBI have been noted both acutely (for complicated and uncomplicated mTBI) and up to ∼6 years post-injury for studies where the distinction between complicated and uncomplicated mTBI was not drawn (Stulemeijer et al., 2010; Konrad et al., 2011). Whereas auditory verbal episodic memory (Halgren et al., 2011) typically improves in individuals with mTBI within a year post-injury, the performance of individuals with complicated mTBI (including individuals with positive findings on CT and/or MRI scans) typically worsens within this time interval (Tayim et al., 2016). In a sample of mixed TBI severities, semantic memory improved over 30 years, with younger age at injury being associated with greater improvement (Himanen et al., 2006). Furthermore, semantic memory may be less impaired in younger patients with a remote mTBI than episodic memory is (Wammes et al., 2017). Unsurprisingly, upon utilizing MRI to study an mTBI cohort ∼30 years post-injury, Himanen et al. (2005) found that poorer episodic memory performance was significantly associated with bilateral volumetric reductions in the hippocampus and with lateral ventricle volume increases. Finally, one dMRI study of adolescents with mTBI found a significant association between reduced WM integrity of the left cingulum bundle and poorer episodic memory performance (Wu et al., 2009). The involvement of the cingulum bundle here is to be expected, given that this structure has been linked to episodic memory performance and to the integration of certain visceral and affective processes which may aid episodic memory consolidation (Lockhart et al., 2012; Bubb et al., 2018).

### Implicit Learning

This review identified very few studies assessing implicit learning after TBI. Three such studies found no impairment of either immediate or delayed implicit learning after closed head TBI, suggesting that this L&M subdomain can remain intact or little affected post-injury (McDowall and Martin, 1996; Schmitter-Edgecombe, 1996; Shum et al., 1996). Because the distinction between complicated and uncomplicated mTBI was not drawn in these investigations, insights on this distinction are not offered by these studies. However, one study involving (A) two tasks measuring non-declarative/implicit memory (i.e., a perceptual priming task and a conceptual priming task) and (B) one declarative memory task found that TBI participants exhibited impairment only during the declarative and conceptual priming tasks (Vakil and Sigal, 1997). This study’s results suggest that perceptual priming may be spared after TBI and emphasize that assessment methodology is critical for the accurate evaluation of implicit memory. When learning new skills, TBI participants exhibited implicit memory impairment during conceptual tasks which typically activate the frontal lobe (e.g., the serial reaction time task and the Tower of Hanoi puzzle task). By contrast, mTBI participants showed no impairment during tasks involving only relatively modest frontal lobe recruitment (e.g., search-detection tasks), although they did have slower response times than HCs (Vakil, 2005; Vakil and Lev-Ran Galon, 2014). Further research is needed to integrate neuroimaging with the assessment of implicit learning after mTBI and to establish whether neuroimaging measures can clarify the precise conditions under which implicit learning can be spared by injury.

## Executive Function

According to the DSM-5, the cognitive domain of EF includes the subdomains of planning, decision-making, working memory, feedback response, inhibition and flexibility (Sachdev et al., 2014). Impairments of overall EF performance have been recorded acutely, up to ∼6 years post-injury after mTBI and up to ∼10 years after TBI of mixed severity, no distinction between complicated and uncomplicated mTBI being drawn (Draper and Ponsford, 2008; Konrad et al., 2011; Rabinowitz and Levin, 2014; McInnes et al., 2017). After mTBI, overall EF was found to improve within the first ∼6 months post-injury (Kwok et al., 2008; Veeramuthu et al., 2015). Schiehser et al. (2011) found that, in mild-to-moderate TBI, the best predictors of overall EF performance were self-reported PCDSs, even after controlling for participants’ effort on tasks. Thus, PCDSs—whether self-reported or independently assessed—should be accounted for when assessing EF after mTBI. Nathan et al. (2012) found that a history of mTBI was associated with abnormal rs-FC of the right thalamus, whereas overall EF was significantly associated with the rs-FC of the left thalamus. Some studies utilizing dMRI (Lipton et al., 2009; Zappalà et al., 2012) indicate that TBI-related EF deficits—including deficits of working memory, reasoning, set-shifting, linguistic and visuospatial abilities—are tied to damage along the association and projective connections of dorsolateral PFC. Sorg et al. (2014) found that mTBI participants who exhibited chronic EF deficits also demonstrated significant reductions in the integrity of WM linking PFC to the rest of the brain, of the corpus callosum and of the cingulum bundle. Such changes were more common in mTBI participants who had experienced LOC at the time of injury. EF is frequently affected in TBI patients who go on to develop post-traumatic epilepsy (Irimia, 2005; Lima et al., 2006; Irimia et al., 2013a, b; Irimia and Van Horn, 2015a).

### Planning

Although mTBI studies examining planning are not abundant, available evidence indicates that this subdomain can be impaired acutely, up to ∼5 months post-mTBI and up to ∼9 months after msTBI (Bar-Haim et al., 2009; Shum et al., 2009; Rabinowitz and Levin, 2014). Impairment severity may be dependent on task complexity; for example, Shum et al. (2009) found that TBI participants were impaired only on the most difficult sections of the Tower of London test and that they did not exhibit impairment on easier sections. On the other hand, some studies (Kraus et al., 2007; Kumar et al., 2013) have found no mTBI-related impairment of planning, possibly due to methodological differences including different sample demographics, injury mechanisms and neuropsychological assessment strategies. In one study of self-reported neurocognitive symptoms, participants reported poorer planning skills both 2–5 years and 5–10 years after injury, regardless of TBI severity (Ponsford et al., 2013). MRI studies typically report a higher prevalence of planning-related impairments in TBI patients who experienced localized PFC damage (Datta et al., 2009; Shum et al., 2009; Nowrangi et al., 2014). Compared to HCs, chronic severe TBI patients exhibit an increase in (A) planning-related BOLD activations within frontal and parietal lobes, and (B) the size of active brain areas, possibly reflecting compensatory mechanisms (Rasmussen et al., 2006). Another fMRI study of chronic severe TBI found that poor planning performance was associated with reduced task-related activation of the left dorsolateral PFC and of the ACC (Cazalis et al., 2006). Miles et al. (2008) identified a significant correlation between a quantitative measure of planning and the dMRI-derived integrity of the centrum semiovale, of the genu and splenium of the corpus callosum, and of the posterior limb of the internal capsule ∼6 months post-TBI, whereas no such correlation had been detected acutely.

### Decision-Making

Deficits of decision-making have been noted after mTBI (no distinction between complicated and uncomplicated cases) as well as msTBI both acutely and up to ∼5 years post-injury (Cotrena et al., 2014). One large-scale, case-control survey found that a history of TBI was significantly associated with increased risk for subsequent problematic gambling, likely due to impaired decision-making, and to subsequent impulsivity; this association was found to be most prevalent in males aged 35–64 (Bhatti et al., 2019). It has also been suggested that poor decision-making after TBI may be mediated by impaired mechanisms for fear recognition. Specifically, Visser-Keizer et al. (2016) found that chronic TBI victims exhibited impaired decision-making and emotion recognition, and that poorer fear recognition was significantly associated with worse task strategy and with more risk-taking behavior. It is unclear whether poorer decision-making after TBI is linked to the altered structure of specific brain regions, although it has been confirmed that such impairments are not limited only to patients with frontal lobe lesions (Levine et al., 2005; MacPherson et al., 2009; Cotrena et al., 2014). Levin et al. (2010) utilized dMRI to study blast-injured veterans with chronic mild-to-moderate TBI and found a significant correlation between poorer decision-making and lower WM integrity along connections between prefrontal regions and both temporal and occipital regions (i.e., the right uncinate fasciculus, the right inferior fronto-occipital fasciculus and the posterior limb of the right internal capsule).

### Feedback Response

Although there has been little research on feedback response after TBI, a few studies which included feedback scores as part of their reported psychometrics suggest that individuals with a history of mTBI are unimpaired on scores reflecting feedback utilization efficacy (Schmidt et al., 2011; Kumar et al., 2013) although studies do not distinguish between complicated and uncomplicated mTBI. Kumar et al. (2013) found no differences between HCs and sub-acute mTBI participants on the Wisconsin Card Sorting Test (WCST) measures of perseverative response or perseverative errors, which suggests unhindered incorporation of feedback into performance. Further support for this hypothesis is provided by Schmidt et al. (2011), who found feedback-based therapy to be modestly effective in improving SA after TBI.

### Working Memory

Impairment of working memory (both visual/spatial and verbal) has been noted after mTBI both acutely and up to ∼8 years post-injury (Konrad et al., 2011; Kumar et al., 2013) with no distinction being drawn between complicated and uncomplicated mTBI. By contrast, some studies have found no impairment in working memory performance after either acute or chronic mTBI, possibly for methodological reasons involving different approaches to neuropsychological assessment and to patient sampling and/or due to the lack of distinction between complicated and uncomplicated mTBI (Johansson et al., 2009; Chen et al., 2012; Zhou et al., 2013). Working memory performance after TBI may also depend upon task complexity and injury severity. For example, individuals with TBI perform worse on tasks requiring advanced cognitive load (e.g., dual task paradigms) compared to easier tasks, and those with msTBI perform worse than those with mTBI (McAllister et al., 2006). Among the very few longitudinal studies of working memory changes after TBI, that of Sanchez-Carrion et al. (2008) found improvements of performance on an n-back task after chronic severe TBI across a 6-month interval. Utilizing fMRI to compare mTBI patients to HCs, McAllister et al. (2001) found that (A) during low cognitive load, the patients’ patterns of fMRI activation were similar to those of HCs, (B) during moderate cognitive load, the patients exhibited greater frontoparietal activations bilaterally, and that (C) during high cognitive load, the patients exhibited weaker bilateral frontoparietal activations. The results of McAllister et al. (2001, 2006) both indicate inefficient brain activation patterns after mTBI which, although relatively unimpacted at low cognitive loads, become apparent at higher loads. According to these authors, moderate loads lead to compensatory over-activation and high loads lead to inadequate fMRI activations. Another fMRI study of moderate cognitive load during an n-back task found a significant positive correlation between bilateral frontal and parietal task-related activation and injury severity (Pardini et al., 2010). Finally, dMRI studies have revealed significant positive correlations between the working memory performances of TBI individuals with TAI and the WM integrity of the superior longitudinal fasciculi, corpora callosa, arcuate fasciculi and fornices (Palacios et al., 2011). Supporting this finding, studies of HCs confirmed the association between (A) WM structure within and between the frontal and temporal lobes and (B) working memory performance (Charlton et al., 2010).

### Response Inhibition

Impairment of response inhibition has been noted acutely, up to ∼2.3 years after mTBI and up to ∼5.7 years after TBIs of mixed severity (Dimoska-Di Marco et al., 2011; Xu et al., 2017), no distinction being drawn between complicated and uncomplicated mTBI. Nevertheless, a large meta-analysis of 41 studies found no relationship between inhibition performance and TBI severity (Dimoska-Di Marco et al., 2011). However, the same meta-analysis did find a significant relationship between longer time since injury and improved response inhibition. In an acute mTBI sample, Dall’acqua et al. (2016) used MRI to identify a positive correlation between bilateral frontal volume reductions and performance on a response inhibition task. By studying BOLD signals recorded during a choice reaction task, Xu et al. (2017) found that chronic mTBI participants exhibited a brain activation pattern in the cerebello-thalamo-cortical network which was reversed compared to that of HCs. Specifically, whereas the task’s Go condition was associated with significantly weaker activation of this network in the mTBI group, its Switch condition was linked to significantly stronger activation in mTBI patients. The Switch condition requires greater inhibitory control, and mTBI subjects’ performance was poorer than that of HCs. By contrast, the Go condition does not require more inhibitory control and there were no differences in performance between groups during this condition. These results suggest the presence of a response inhibition deficit following mTBI. Fischer et al. (2013) confirmed the reverse brain activation pattern observed by Xu et al. (2017) when studying chronic, mild-to-moderate TBI; these authors identified bilateral BOLD signal increases in the caudate nuclei and in the left superior temporal, inferior temporal and cerebellar cortices, especially in relation to failures to inhibit a response.

### Cognitive Flexibility

Deficits of cognitive flexibility have been documented ∼2 months post-mTBI by Pang et al. (2016), and ∼4.7 years after msTBI by Leunissen et al. (2014) although very few other studies could be located. Patients may recover from such deficits; for example, although acute TBI participants’ task switching (cognitive flexibility) was consistently poorer than that of HCs, patients improved in this respect over the 1st month after injury (Mayr et al., 2014). Leunissen et al. (2014) found that, ∼4.7 years post-TBI, the volumes of cortical regions with connections to prefrontal or to rostral motor areas were inversely correlated with task switching performance, which highlights the importance of fronto-striato-thalamic circuits. The authors also found that task-switching performance after TBI was best predicted by the integrity of WM connections between the superior frontal gyrus (pre-supplementary motor area) on the one hand and the putamen, caudate nucleus as well as thalamus, on the other hand.

## Language

According to the DSM-5, the cognitive domain of language includes subdomains corresponding to both expressive language (naming, word-finding, fluency, grammar and syntax) and receptive language (Sachdev et al., 2014). Language domain deficits have been noted during both acute and chronic mTBI, up to ∼3.3 years post-injury (King et al., 2006; Rapoport et al., 2006; Galetto et al., 2013) no distinction between complicated and uncomplicated mTBI being drawn. According to one meta-analysis, mTBI participants exhibited better language performance ∼3 months-post-injury compared to the acute stage, which illustrates how language can improve over time (Belanger et al., 2005). Interestingly, language deficits observed after mTBI (e.g., global incoherence, inaccuracy of information, disruption of utterances) could be consequences of other high-order cognitive impairments—such as slower processing speed, inefficient attentional processing, EF disruption and poor memory encoding—rather than manifestations of true language deficits (Barwood and Murdoch, 2013; Galetto et al., 2013). However, caution should be exerted when drawing any conclusions pertaining to this topic due to the relative paucity of adequately powered studies investigating language after mTBI.

### Word-Finding and Naming

Despite the separation of these two subdomains under the DSM-5 classification system, naming is considered a type of word finding, by means of which the latter is often assessed (Rohrer et al., 2007). Disruptions of the ability to name objects presented visually is among the most common language-related complaints after TBI in general, and mTBI in particular (King et al., 2006; Kennedy et al., 2009). Naming deficits have been observed after mTBI both acutely and up to ∼1.2 years post-injury (King et al., 2006; Miotto et al., 2010), no distinction between complicated and uncomplicated mTBI being drawn. King et al. (2006) found that acute mTBI patients exhibited impairment in confrontational naming but not in natural discourse naming, which highlights the possibility that slight language deficits apparent on psychometric tests may not be readily detectable in everyday life. These authors also concluded that the most common naming error among mTBI participants involves latency (i.e., the time taken to respond to a stimulus). It is possible that younger age at injury is associated with better naming performance after mTBI; Li et al. (2017) found that performance on the Boston Naming Test (the BNT, a commonly used naming test) was better in individuals who had suffered a TBI before the age of 22 rather than after. Based on clinical lesion data, Miotto et al. (2010) concluded that chronic mild-to-moderate TBI patients who were impaired on a naming task were most likely to have a frontotemporal lesion.

### Verbal Fluency

Studies usually assess two types of verbal fluency: semantic (production of words of a single category, such as vegetables) and phonemic (production of words which start with a specific letter). Impairment of verbal fluency has been found in both complicated and uncomplicated mTBI during the acute stage, as well as up to ∼2 years post-injury, with improvements over time (Wallesch et al., 2001; Belanger et al., 2005; Zakzanis et al., 2011; Croall et al., 2014). Although semantic fluency may be more impaired than phonemic fluency after TBI, one meta-analysis of 30 studies found comparable deficits in both types of fluency, suggesting an underlying EF deficit (Henry and Crawford, 2004). Nevertheless, Wallesch et al. (2001) found GCS-measured TBI severity to be significantly and positively correlated with semantic fluency 5–10 months post-injury, but not with phonemic fluency. Thus, it is unclear whether semantic fluency is more vulnerable to mTBI than phonemic fluency. Interestingly, both types of fluency impairment are usually associated with TBI-related pathology of the frontal and temporal lobes (Wallesch et al., 2001; Henry and Crawford, 2004; Zakzanis et al., 2011). One DTI study found that acute verbal fluency deficits in mild-to-moderate TBI patients were negatively correlated with WM integrity and positively correlated with radial and axial diffusivity throughout the brain, but especially within the ascending fibers of the corpus callosum in the left hemisphere (Croall et al., 2014).

### Grammar and Syntax

Grammar and syntax may not be affected considerably in the spontaneous speech of TBI patients, as recent studies have found no impairments of such abilities in either mTBI (∼3.3 years post-injury) or moderate TBI (∼1.9 years post-injury), whereas severe TBI patients had somewhat worse performance (∼5.5 years post-injury) (Galetto et al., 2013; Marini et al., 2014, 2017), no distinction being drawn between complicated and uncomplicated mTBI. Although TBI-related syntactic deficits have been noted, such deficits may be the consequence of a primary semantic deficit or, alternatively, could be characteristic of specific samples (e.g., of patients with both TBI *and* aphasia) (Coelho et al., 2005).

### Receptive Language

In one of the few adult studies available on receptive language deficits after TBI (Chabok et al., 2012), ∼65% of a mixed-severity TBI sample were found to exhibit acute language deficits. Of these, ∼38% exhibited impairments of auditory story comprehension, a measure of receptive language. The same study found that both moderate and severe injuries as well as fronto-temporal lesions were risk factors for language deficits, including comprehension difficulties. Menon et al. (1993) found that, although receptive language performance improved post-TBI, this subdomain was more impaired after severe than after mild-to-moderate injury. Receptive language deficits were found to be highly correlated with impairments of both short- and long-term memory as well as with EF impairments, indicating that altered language comprehension after TBI may stem from primary deficits in other cognitive domains (Vukovic et al., 2008). Finally, while investigating older adults with chronic mTBI, Barwood and Murdoch (2013) found specific deficits related to (A) the comprehension of ambiguous sentences and temporal structures, (B) inference construction based on listening comprehension, as well as to (C) recognition and expression of words’ semantic properties.

## Perceptual-Motor Function

According to the DSM-5, perceptual-motor function includes as subdomains visual perception, visuo-constructional reasoning, and perceptual-motor coordination (Sachdev et al., 2014). Perceptual-motor dysfunction can occur frequently in TBI patients (Heitger et al., 2006). Because researchers do not typically assess perceptual-motor function as an entire domain, what follows is an examination of TBI studies on its subdomains.

### Visual Perception

Visual perception is an overarching term referring to (A) primary visual detection (which relies on visual acuity, visual fields, saccades, convergence, etc.) and (B) higher-level visual processing (which involves visual scanning, recognition of faces and objects, visual memory, visual attention, etc.). Thus, mTBI-related dysfunction of visual perception (e.g., reading difficulty) might stem from impaired visual detection (due to dysfunction of processes like saccades and convergence, which involve visual pathways between the retina and visual cortex), or from impaired visual processing (due to damage to visual cortex and associated cortices); both scenarios have been reported after mTBI (Magone et al., 2014; Barnett and Singman, 2015). Impairments of visual perception (i.e., deficits of detection and processing) after mTBI have been recorded acutely and up to ∼1.5 years post-injury (up to ∼4.2 years post-injury for visual detection deficits alone) (Magone et al., 2014; Alnawmasi et al., 2019), no distinction being drawn between complicated and uncomplicated mTBI. In a retrospective study of blast-induced mTBI, visual complaints were reported by 68% of participants, the most common being photophobia and reading difficulties (Magone et al., 2014). About 25% of the sample had been diagnosed with convergence insufficiency and ∼23% with accommodative insufficiency, suggesting damage to visual detection pathways. Such visual detection impairments, including visual field loss, have higher prevalence in msTBI than in mTBI, and can be detected after both blast-induced (military) and non-blast-related (civilian) mTBI (Capó-Aponte et al., 2017; Merezhinskaya et al., 2019). Commonly reported deficits of higher-order visual processing include impairments related to form recognition, motion perception, and figure/ground discrimination (Ciuffreda et al., 2016; Alnawmasi et al., 2019). Because there are hardly any reports of statistically significant associations between time since injury and visual perception performance, such deficits may remain stable over relatively long periods (Alnawmasi et al., 2019).

### Visuo-Constructional Reasoning

Visuo-construction has been found to be mildly impaired both acutely and up to ∼1 year after complicated mTBI (Kashluba et al., 2008). However, no difference in visuo-constructional ability has been detected between mTBI and moderate TBI either acutely or at ∼1 year post-injury. The importance of accounting for participants’ test effort was highlighted by Aguerrevere et al. (2014). These authors found no visuo-constructional deficits in mTBI participants who had invested an expected amount of effort while being tested, although no distinction was drawn between complicated and uncomplicated mTBI. The authors found moderate deficits in mTBI participants with poor investment of effort and in msTBI participants. Longitudinally, the performance of patients with TBIs of various severities on a visuo-constructional task was found to improve significantly from 1 to 5 years post-injury, with only 1.3% of the sample still being impaired after 5 years (Millis et al., 2001). Upon utilizing fMRI to investigate sub-acute mTBI, Tang et al. (2011) found a significant inverse relationship between bilateral rs-FCs involving the thalamus and performance on the Rey Complex Figure Test (RCFT, commonly used to assess visuospatial and visual memory skills). Upon leveraging dMRI to study sub-acute mild-to-moderate TBI, Kumar et al. (2009) found a significant, positive correlation between performance on the Block Design Test (BDT, commonly used to assess visuo-construction) and WM integrity in the genu of the corpus callosum. This study also found a positive linear relationship between performance on the BDT and axial diffusivity within the genu and splenium of the corpus callosum. The involvement of the corpus callosum may be due to its role in the inter-hemispheric transfer of visuomotor information, which is required by visuo-constructional tasks (Schulte et al., 2005). On the other hand, the thalamus is intimately involved in EF, memory and attention processing, but not in visuo-constructional reasoning (Tang et al., 2011). These neuroimaging results, however, are relatively novel and thus require replication.

### Perceptual-Motor Coordination

Deficits of perceptual-motor (mostly visual) coordination after mTBI have been reported both acutely and up to ∼1 year post-injury (Heitger et al., 2006), no distinction being drawn between complicated and uncomplicated mTBI. Acute mTBI has been associated with (A) prolonged latencies and decreased accuracy of saccades, (B) greater directional errors, (C) impaired sinusoidal smooth pursuit involving longer reaction times during arm movements, and (D) poorer upper-limb visuomotor tracking performance marked by lower arm speed and accuracy. These deficits were reported to improve within 1 year post-injury. Heitger et al. (2009) recorded similar oculomotor deficits ∼3 to ∼5 months post-mTBI, which could not be explained by group differences related to PCDSs or by intellectual ability. The study found a significant correlation between increased oculomotor deficits, on the one hand, and both more self-reported PCSs as well as poorer quality of life, on the other hand. Interestingly, there was no correlation between oculomotor deficits and neuropsychological measures. Perceptual-motor coordination (as measured by oculomotor performance) has been suggested to remain relatively stable between the ages of 16 and 70 (Heitger et al., 2009).

Ventura et al. (2016) found that, relative to HC participants, acute mTBI patients’ impaired oculomotor performance was linked to increased BOLD activations in (A) the cerebellum and visual cortex (during anti-saccades and self-paced saccades), (B) dorsolateral PFC, bilaterally (during self-paced saccades), and (C) the left hippocampus, right lingual gyrus, left precentral gyrus, cerebella, left frontal eye fields, precunei and brainstem during memory-guided saccades. Similar outcomes were found 30 days post-injury, albeit fMRI activations were weaker. These results may suggest a compensatory increase in brain activation after mTBI. The authors also found that chronic mTBI patients’ oculomotor deficits were correlated with poor WM integrity (as measured by fractional anisotropy) in the right anterior corona radiata, left superior cerebral peduncle and genu of the corpus callosum. The involvement of these areas in perceptual-motor coordination after mTBI is plausible given their established importance in information integration, in the refinement of motor movements and in the inter-hemispheric transfer of visuomotor information (Schulte et al., 2005; Han et al., 2010; Kwon et al., 2011). However, further research is needed to confirm and to further establish the neuroimaging correlates of perceptual-motor coordination after mTBI.

## Neuropsychological Assessments

Tables 1–5 list all reviewed neuropsychological tests by cognitive domain, whereas Supplementary Table 1 reproduces this information as one single table because this format allows the reader to compare the utility of various tests across domains. Table 6 lists all abbreviations utilized in the text, including this section.

**TABLE 1:** Psychometric instruments for the assessment of the cognitive domain of attention, including overall attention and its subdomains, i.e., sustained attention, divided attention, selective attention and processing speed.

	Overall attention	Sustained attention	Divided attention	Selective attention	Processing speed
SDMT	✓		✓		✓
DS	✓				✓
DSCT	✓				✓
TMT-A	✓				✓
TMT-B	✓				✓
TAP	✓		✓		
CPT	✓			✓	
ANT	✓			✓	
SART	✓	✓			
PVSAT	✓				✓
PASAT	✓	✓			✓
MCT		✓			
CRTT		✓			✓
DVT		✓			
DTT			✓		
CMT			✓		✓
SCWT					✓
NST				✓	
CSAT				✓	

**TABLE 2:** Psychometric instruments for the assessment of the cognitive domain of learning and memory, including overall learning and memory and its subdomains, i.e., free recall, cued recall, recognition memory, episodic memory, semantic memory, and implicit learning.

	Learning and memory	Free recall	Cued recall	Recognition memory	Episodic memory	Semantic memory	Implicit learning
VFT						✓	
RAVLT	✓	✓		✓			
CVLT-II	✓	✓	✓	✓			
MC 1 and 2	✓						
WMT	✓	✓	✓	✓			
DPT	✓						
WMS	✓				✓		
IED	✓						
RCFT	✓						
FWM	✓						
BSRT		✓					
PN and SM						✓	
ITTI							✓
SRTT							✓
TOHT							✓

**TABLE 3:** Psychometric instruments for the assessment of the cognitive domain of executive function, including overall executive function and its subdomains, i.e., planning, decision-making, feedback response, working memory, response inhibition and cognitive flexibility.

	Executive function	Planning	Decision-making	Feedback response	Working memory	Response inhibition	Cognitive flexibility
DS					✓		
TMT-A							✓
TMT-B	✓						✓
CPT						✓	
SART	✓					✓	
PASAT					✓		
SCWT						✓	✓
VFT	✓						
IED							✓
HSCT	✓						
BSAT	✓						
COWAT	✓						✓
PMT	✓						
TOL	✓	✓					
WCST	✓			✓			✓
DKEFS	✓						
NAB	✓						
BADS		✓					
PF A and B		✓					
IGT			✓				
N-Back					✓		
G/N and SST						✓	
LGST							✓
SP					✓		

**TABLE 4:** Psychometric instruments for the assessment of the cognitive domain of language, including overall language and its subdomains, i.e., word-finding (naming), verbal fluency, grammar and syntax, and receptive language.

	Language	Word-finding (naming)	Verbal fluency	Grammar and syntax	Receptive language
DS	✓				
VFT	✓		✓		
COWAT			✓		
WCST	✓				
RIDR	✓				
NSTT	✓			✓	
BNT		✓			
AWF		✓			
TWFD		✓			
RFFT			✓		
AAT				✓	
PPVT					✓
TT					✓
ACT					✓

**TABLE 5:** Psychometric instruments for the assessment of the cognitive domain of perceptual-motor function, including overall perceptual-motor function and its subdomains, i.e., visual perception, visuo-construction and perceptual-motor coordination.

	Perceptual-motor function	Visual perception	Visuo-construction	Perceptual-motor coordination
RCFT			✓	
CP 1		✓		
BDT			✓	
LOJT			✓	
HVOT			✓	
BVRT			✓	
CP 2				✓
CP 3				✓

*There are no entries in the perceptual-motor function column because the listed instruments assess cognitive subdomains rather than the overall domain of perceptual-motor function.*

**TABLE 6:** Abbreviations used throughout the text.

AAT	Aachen Aphasia Test
ACC	anterior cingulate cortex
ACT	Auditory Comprehension Tests
AD	Alzheimer’s disease
ANT	Attention Network Task
AWF	Test of Adolescent/Adult Word Finding
BADS	Behavioral Assessment of the Dysexecutive Syndrome
BDT	Block Design Test
BNT	Boston Naming Test
BOLD	blood oxygen level-dependent
BSAT	Brixton Spatial Anticipation Test
BSRT	Buschke Selective Reminding Test
BVRT	Benton Visual Retention Test
CBF	cerebral blood flow
CMT	custom-made tests
COWAT	Controlled Oral Word Association Test
CP 1	computerized paradigms involving matching, visual search, reading comprehension, visual recognition, figure/ground discrimination and motion perception
CP 2	computerized paradigms involving reflexive saccades, anti-saccades, memory-guided saccade sequences, self-paced saccades and both sine and random oculomotor smooth pursuits
CP 3	computerized paradigms involving an output accessory
CPT	Continuous Performance Test
CRTT	Simple Choice Reaction Time Task
CSAT	Complex Selective Attention Task
CT	computed tomography
CVLT II	California Verbal Learning Test II
D-KEFS	Delis–Kaplan Executive Function System
DMN	default mode network
dMRI	diffusion magnetic resonance imaging
DPT	Doors and People Test
DS	Wechsler Adult Intelligence Scale III Digit Span
DSCT	Wechsler Adult Intelligence Scale III Digit Symbol Coding Task
DSM-5	Diagnostic and Statistical Manual of Mental Disorders 5
DTI	diffusion tensor imaging
DTT	Dual-Task Tests
DVT	Digit Vigilance Test
EF	executive function
FC	functional correlation
fMRI	functional magnetic resonance imaging
FWM	Four Word Short-Term Memory Test
G/N and SST	Go/No-go and Stop-Signal Task
GAD	generalized anxiety disorder
GCS	Glasgow Coma Score
GM	gray matter
HC	healthy control
HSCT	Hayling Sentence Completion Test
HVOT	Hooper Visual Organization Test
IED	Intra-Extra Dimensional Set Shift Test (part of the Cambridge Neuropsychological Test Automated Battery)
IGT	Iowa Gambling Task
ITTI	Implicit Test of Tachistoscopic Identification
L&M	learning and memory
LGST	Local-Global Switching Tasks
LOC	loss of consciousness
LOJT	Line Orientation Judgment Test
MC 1 and 2	Memory Cabinet 1 and 2
MCI	mild cognitive impairment
MCT	Monotone Counting Test
mPFC	medial prefrontal cortex
MRI	magnetic resonance imaging
msTBI	moderate-to-severe traumatic brain injury
mTBI	mild traumatic brain injury
NAB	Neuropsychological Assessment Battery
N-Back	N-Back Working Memory Task
NST	Numeric Stroop Test
NSTT	Narrative Story-Telling Tests
PASAT	Paced Auditory Serial Addition Test
PCC	posterior cingulate cortex
PCDS	post-concussive depressive symptom
PCS	post-concussive symptom
PET	positron emission tomography
PF A and B	Prioritization forms A and B
PFC	prefrontal cortex
PMT	Porteus Maze Test
PN and SM	Picture Naming and Semantic Priming tests
PPVT	Peabody Picture Vocabulary Test
PTA	post-traumatic amnesia
PTSD	post-traumatic stress disorder
PVSAT	Paced Visual Serial Addition Test
RAVLT	Rey Auditory Verbal Learning Test
RCFT	Rey Complex Figure Test
RFFT	Ruff Figural Fluency Test
RIDR	Rey’s 15-word Immediate and Delayed Recall Test
RME	Reading the Mind in the Eyes Test
rs	resting state
SA	self-awareness
SART	Sustained Attention to Response Task
SCWT	Stroop Color-Word Test
SDMT	Symbol Digit Modalities Test
SP	Sternberg Paradigm for Verbal and Visuo-spatial Working Memory
SRTT	Serial Reaction Time Task
TAI	traumatic axonal injury
TAP	Test of Attentional Performance
TBI	traumatic brain injury
TMT-A	Trail-Making Test A
TMT-B	Trail-Making Test B
TOHT	Tower of Hanoi Task
TOL	Tower of London Test
ToM	theory of mind
TT	Token Test
TWFD	Test of Word Finding in Discourse
US	United States
VFT	verbal fluency tests
WAIS	Wechsler Adult Intelligence Scale
WASI	Wechsler Abbreviated Scale of Intelligence
WCST	Wisconsin Card Sorting Test
WM	white matter
WMS	Wechsler Memory Scale
WMT	Word Memory Test

*Following convention, all names of psychometric tests, tasks, batteries, forms and scales are capitalized.*

For the assessment of overall attention (Table 1), commonly used neuropsychological tests include the Symbol Digit Modalities Test (SDMT), the Wechsler Adult Intelligence Scale III Digit Span (WAIS III DS), the WAIS III Digit Symbol Coding Task (DSCT), the Trail-Making Test A (TMT-A), the Trail-Making Test B (TMT-B), the Test of Attentional Performance (TAP), the Continuous Performance Test (CPT), the Attention Network Task (ANT), the Sustained Attention to Response Task (SART), the Paced Visual Serial Addition Test (PVSAT), the Paced Auditory Serial Addition Test (PASAT), etc. Neuropsychological tests previously used to assess sustained attention in TBI participants include the SART, the Monotone Counting Test (MCT), the simple choice reaction time task (CRTT), the PASAT and the Digit Vigilance Test (DVT). Neuropsychological tests used to assess divided attention include the SDMT, dual-task tests (DTTs), the TAP and custom made tests (CMTs). Neuropsychological tests used for selective attention in the studies reviewed include the Attentional Network Task (ANT), the Numeric Stroop Test (NST), the Complex Selective Attention Task (C-SAT) and the CPT. The neuropsychological tests used to assess processing speed include the TMT-A, TMT-B, simple reaction time tests, the SDMT, the WAIS III DS and DSCT, custom visual and tactile reaction time tasks, the Stroop Color-Word Test (SCWT), the DSCT, PVSAT and PASAT.

Tests commonly used to assess overall L&M after TBI (Table 2) include the Rey Auditory Verbal Learning Test (RAVLT), the California Verbal Learning Test (CVLT-II), the Memory Cabinet (MC) 1 and 2, the Word Memory Test (WMT), the Doors and People Test (DPT), the Wechsler Memory Scale (WMS), the Cambridge Neuropsychological Test Automated Battery, the Rey Complex Figure Test (RCFT) and the Four Word Short-Term Memory (FWM) Test. Tests used to assess free and cued recall are usually composite subtests or sub-trials of established memory batteries. For free recall, these include sub-trials of the RAVLT and WMT, the Buschke Selective Reminding Test (BSRT), and CVLT-II subtests; for cued recall, the WMT-Paired-Associations sub-trial and CVLT-II subtests are used frequently. Subtests are also used to assess recognition memory after TBI (e.g., the WMT-Multiple-Choice subtest, RAVLT sub-trials and CVLT-II sub-trials). Whereas episodic memory is typically assessed using the WMS, common semantic memory tests include verbal fluency tests (VFTs), picture naming and semantic priming (PM and SM) tests. Finally, tests of implicit memory include the Implicit Test of Tachistoscopic Identification (ITTI), the Serial Reaction Time task (SRTT) and the Tower of Hanoi task (TOHT).

The assessment of the overall EF domain after TBI (Table 3) typically relies on the TMT-B, Hayling Sentence Completion Test (HSCT), on semantic and lexical VFTs, SART, Brixton Spatial Anticipation Test (BSAT), Controlled Oral Word Association Test (COWAT), Porteus Maze Test (PMT), PMT Vineland Revision, TOL II, WCST (64-card version), Wechsler Abbreviated Scale of Intelligence (WASI) Matrix Reasoning subtest, Delis–Kaplan Executive Function System (D-KEFS), and on the Neuropsychological Assessment Battery (NAB) Word Generation and Mazes modules. To assess the subdomain of planning, the TOL test, Behavioral Assessment of the Dysexecutive Syndrome (BADS), and the Prioritization Forms (PFs) A and B are frequently used. Assessment of decision-making post-TBI has relied almost exclusively on the Iowa Gambling task (IGT) and on modified versions of it. The few studies which assessed feedback responses relied on the WCST. Working memory has been evaluated using the Sternberg Paradigm (SP) for verbal and visuo-spatial working memory, the DS task, the *n*-back working memory task and several PASAT subtests. Inhibition has been typically assessed using the go/no-go task, stop-signal task (G/N and SST, respectively), SCWT, SART and CPT. Finally, cognitive flexibility has been measured using the SCWT, TMT-A and -B, WCST, COWAT, the intra-extra dimensional (IED) set shift test (part of the Cambridge Neuropsychological Test Automated Battery) and using custom local-global switching tasks (LGSTs).

Neuropsychological tests most commonly employed to assess the overall language domain (Table 4) after TBI included VFTs, the WAIS III DS, Rey’s 15-word Immediate and Delayed Recall Test (RIDR), the WCST (perseverative and non-perseverative errors), and a variety of narrative story-telling tests (NSTTs). To assess word finding and naming, researchers typically used the BNT, the Test of Adolescent/Adult Word Finding (AWF), and the Test of Word Finding in Discourse (TWFD). Fluency was often assessed using the COWAT, the Ruff Figural Fluency Test (RFFT), VFTs and the FAS test. The assessment of grammar and syntax was accomplished with the Aachen Aphasia Test (AAT) subtests and NSTTs based upon pictures from the Western Aphasia Battery. Finally, receptive language has been assessed using the Peabody Picture Vocabulary test (PPVT), the Token Test (TT) and various auditory comprehension tests (ACTs).

Tests commonly employed to assess the subdomain of visual perception after TBI (Table 5) were usually custom computerized tests involving paradigms such as matching, visual search (often assessed through the Weinberg Visual Cancelation Test), reading comprehension, visual recognition, figure/ground discrimination and motion perception. Visuo-constructional reasoning was typically assessed using the BDT, simple copy tests, complex copy tests (especially the RCFT), draw-to-command tasks [e.g., the Line Orientation Judgment Test (LOJT)], the Hooper Visual Organization Test (HVOT) and the Benton Visual Retention Test (BVRT). Tests of assessing perceptual-motor coordination after TBI often vary depending on the ability being studied. For instance, oculomotor assessment is typically done using computerized paradigms, involving reflexive saccades, anti-saccades, memory-guided saccade sequences, self-paced saccades and both sine and random oculomotor smooth pursuits. Limb coordination tests used after TBI typically utilize computerized paradigms involving an output accessory (such as a steering wheel which controls the movements of an arrow on the computer screen).

Despite the abundance of psychometric instruments, there are potential problems which may arise with their use. Firstly, many cognitive assessments vary considerably in their capacity to detect mTBI impairments (Karr et al., 2013). For example, in a study by Draper and Ponsford (2008) the SDMT and DSCT uncovered overall attention deficits in TBI participants, whereas the TMT-A, DS and the SART did not. In the same study, the RAVLT and the DPTs uncovered overall memory deficits in TBI, whereas the Shapes and Names tests did not. Similarly, the HSCT and SART uncovered overall EF deficits in TBI, but the PMT, BSAT, COWAT and TMT-B did not. To provide more nuanced information on the advantages and limitations of psychometric batteries, future studies should strive to report detailed findings on TBI participants’ performances on each cognitive subdomain assessed by each instrument. One motivation is that, whereas TBI-related impairments may not be reflected by participants’ overall scores, such impairments may be highlighted by test sub-scores. Furthermore, simple tasks are frequently administered to assess complex cognitive processes in TBI patients. For example, simple reaction time tasks like the TMT-A are often used to assess complex overall attention, although such tasks are suitable for the sole assessment of processing speed (Paré et al., 2009). Another example is the common use of the RAVLT to assess overall memory, although this test focuses solely on the assessment of auditory verbal episodic memory (Magalhães et al., 2012). Similarly, overall language domain assessments frequently involve evaluations of naming abilities (like the BNT), which quantify word finding, but not other language subdomains.

Some studies have used complex tests (Tate et al., 2017) or more than one test (Kraus et al., 2007) to measure overall cognitive domain function after TBI, although this strategy may be of limited benefit because the comprehensive psychometric quantification of entire domains is quite difficult to accomplish. For example, the PASAT—which is commonly used to assess overall attention after TBI—can measure a wider range of abilities, including processing speed, sustained attention, divided attention and working memory (Tate et al., 2017). However, because the PASAT does not directly measure selective attention, this test may not be best for systematic assessment of the overall attention domain. Similarly, Kraus et al. (2007) combined the DS, Spatial Span, TMT-A and CPT to assess overall attention, but the interpretation of their results is limited because none of these instruments measure divided attention. Thus, compared to studies focusing on cognitive subdomains, TBI studies featuring complex tests or combinations of tests to measure cognitive function across an entire domain can rarely be comprehensive in their assessment. Firstly, because psychometric instrument selection constrains which subdomains are studied, misconceptions can arise if researchers use tests which measure only certain subdomains and then use the results of such tests to draw conclusions about an entire domain. Secondly, it is more challenging to relate overall domain (e.g., attention) performance to neuroimaging-based measures rather than to specific subdomains (e.g., selective or divided attention). This is because correlations between measures of overall domain function and neuroimaging metrics can often be relatively non-specific and may additionally be confounded across studies by the fact that distinct studies use different testing batteries and approaches. For example, Little et al. (2014) could identify structural correlates of overall attention across brain regions, although without spatial specificity. By contrast, Zhou et al. (2013) studied specific attention subdomains and could identify specific brain regions whose anatomic changes could be linked to performance within subdomains (e.g., right rostral ACC atrophy correlated with sustained attention). Thus, studying cognitive subdomains may help to characterize the statistical relationships between brain structure and function in ways which are potentially more specific and more replicable. Although such an approach is slowly gaining adoption—especially to test language and perceptual-motor function—it is still far from common.

Ideally, meta-analyses should integrate information on the separate subdomains to paint a comprehensive image of their richness and complexity. This, however, requires addressing the heterogeneity of taxonomies used to classify cognition into domains and subdomains. Even the DSM-5 classification system, as one of the few established systems for classifying cognitive functions, is controversial and limited in scope. For example, in defining L&M, the DSM-5 does not distinguish well between immediate and delayed recall, between verbal and non-verbal/visual memory, or between prospective and retrospective memory. Additionally, the lack of a uniform standard for technical nomenclature among TBI psychologists and psychometricians remains a major obstacle to research synthesis. For example, some studies of specific L&M subdomains do not explicitly mention their names (e.g., semantic memory). In these cases, rather than relying on the terminologies used in studies, one must examine the specific tests used by researchers to determine what is being measured. Thus, researchers should strive to conform to a classification system which is broadly agreed-upon, thereby enabling direct and unhindered comparison of studies.

Even if studies choose to investigate cognitive subdomains systematically and distinctly, problems may arise when using a test which measures multiple abilities. For example, the PASAT–an elaborate test measuring multiple cognitive abilities across several domains–is often used to assess sustained attention in particular (Zhou et al., 2013). To reduce the potential confounds of other domains being examined when evaluating sustained attention using the PASAT, the ability to clearly identify test sections which measure sustained attention in isolation would be of substantial assistance. Another example is the common use of the Stroop Color-Word test to assess inhibition. One large meta-analysis of 41 studies found a non-significant overall effect for this test in TBIs of mixed severities, and reported that the reliability of the test may vary substantially across samples (Dimoska-Di Marco et al., 2011). These findings could either be due to (A) a lack of a TBI-related deficits in interference control (a type of response inhibition) as measured by this test, (B) canceling out of participants’ aptitude in the different abilities measured by this test, or (C) vulnerability of the Stroop test to confounds like poor processing speed, under-arousal or fatigue (Dimoska-Di Marco et al., 2011). A further example is the RCFT, which is often used to assess visuo-constructional reasoning and visual memory (Tang et al., 2011). As in the case of other multifaceted psychometric tests, the precise weighting of each ability tested by the RCFT is not necessarily made clear by its scoring system, such that quantifying and interpreting performance within specific cognitive subdomains can be challenging. Thus, future research should aim to use psychometric approaches which unambiguously delineate the abilities being measured. Additionally, the scoring systems of commonly used tests should be expanded to include distinct scores for all such relevant abilities. Alternatively, new psychometric instruments should be developed in which the loads and scoring schemes for each measured ability are made explicit.

Neuropsychological testing of TBI effects on cognition can often fail to account for both verbal *and* non-verbal elements of the cognitive abilities being measured. For example, whereas commonly used memory assessment batteries focus on verbal memory assessment (e.g., RAVLT, CVLT-II), many do not quantify non-verbal memory function as systematically. An illustration of a favorable approach is provided by the study of Chan (2005) where the SART and the MCT—both of which focus on sustained attention)—are used. Because the SART and the MCT assess visual and auditory components of sustained attention, respectively, the combined use of these two instruments adds another dimension of valuable information to the conventional characterization of sustained attention provided by other tests.

When studies do not capture the multidimensional nature of cognitive domains and the relationships between their subdomains, interpretative challenges may arise. Specifically, impairments on neuropsychological tests after TBI may be due to other more fundamental pathology affecting the cognitive abilities being measured. For example, sustained attention is predicated upon other basic cognitive abilities, including working memory, cognitive control, inhibition, and flexibility (Pontifex et al., 2012). Another example concerns response inhibition deficits, which may be due to poor processing speed, to fatigue or to the arousal state of the subject rather than to interference control *per se* (Dimoska-Di Marco et al., 2011). Similarly, impaired performance on sustained attention tasks can be considerably influenced by fatigue, depressed mood or by sleep alterations, all of which are frequently reported by mTBI patients (Sinclair et al., 2013). Finally, processing speed may explain much of the variance in performance observed on many neurocognitive tests which assess various cognitive functions and which involve a timed component (Mathias and Wheaton, 2007). Thus, caution should be exerted when interpreting the results of such tests to draw conclusions which exclusively concern very specific aspect of cognition.

## Injury Severity and Clinical Factors

Injury whose severity is greater than mild (i.e., msTBI) appears to be predominantly linked to greater and to more persistent cognitive and affective difficulties compared to mTBI. This distinction in the gravity of sequelae across severities can be seen within each of the cognitive domains examined in this review (affect, social cognition, complex attention, learning and memory, executive function, language and perceptual-motor function), and is particularly notable for affect (depression and anxiety) and social cognition (specifically for emotion recognition and self-awareness), although further studies of social cognition post-msTBI are needed to clarify the role of injury severity within this domain. Two potential exceptions to this trend may pertain to (A) ToM performance, for which no reports of injury severity effects have been identified here, and (B) the amount of effort expended by patients during cognitive assessment. Specifically, msTBI has occasionally been linked to a lower likelihood of poor effort on tasks related to neuropsychological testing, which may suggest that accounting for patients’ expended effort is particularly important in mTBI studies. Whenever possible, future research should aim to quantify the relationship between injury severity and the amount of effort expended by patients during their assessment, given that this distinction has not been quantified rigorously and that its confounding effect remains unclear for the assessment of many—if not most—cognitive subdomains (Hinojosa-Rodriguez et al., 2017).

When analyzing differences between TBI severities, factors conventionally categorized as clinical (such as PTA, LOC, GCS and neuroimaging results) are very important when assessing injury impact on cognitive and affective processes (National Academies of Sciences and Engineering, 2019). These clinical factors have been highlighted throughout the review, wherever pertinent data are available. Overall, our findings appear to support the notion that more extensive PTA, longer LOC, lower GCSs and more abnormal neuroimaging findings—all of which are typical of greater TBI severity–are associated with poorer cognition and affect. For example, all clinical factors mentioned above have been linked to poorer sustained attention performance. In the studies reviewed here, GCS is nearly always used to distinguish between patients based on their injury severity (mild, moderate or severe). Because of this, we find GCS to be consistently associated with poorer performance on assessments of cognition and affect across all domains, with the potential exceptions listed in the previous paragraph. As the second most studied clinical factor apart from neuroimaging, PTA is often indicative of decreased performance in many—but not all—domains and subdomains, including attention, memory, L&M (specifically free recall, working memory and recognition memory), EF (specifically response inhibition and cognitive flexibility) and the overarching domain of language. The relation of neuroimaging results to post-traumatic cognition and affect is currently under extensive study (National Academies of Sciences and Engineering, 2019). Although findings of abnormal structural and function have typically been linked to poorer performance, adequate interpretation may depend upon complex and problematic factors like assessment modality, the areas/functions assessed and the researchers’ categorization, taxonomy and conceptualization of cognitive domains/subdomains. Research within this area of study therefore requires further standardization before more adequate or generalizing conclusions can be drawn. Furthermore, a mention of the fact that TBI clinical factors can be interdependent should not be omitted here. For example, longer LOC in mTBI participants exhibiting EF deficits is significantly correlated with WM damage severity, as revealed by neuroimaging. By contrast, many relationships between clinical factors and post-traumatic cognition and affect are still unclear. For example, there is no agreed-upon conclusion pertaining to LOC length effects on selective attention performance post-TBI and additional studies should be undertaken to improve our understanding on this association.

## Limitations and Conclusion

This review summarizes recent literature on mTBI cognitive dysfunction, on its neuroimaging correlates, and on its relation to neuropsychological assessment. Discussions of PCSs are included for each cognitive (sub)domain as a function of age at injury, time since injury and other assessment categories. Neuroimaging studies indicates that, despite substantial research on the relationship between brain structure, brain function and cognition, certain cognitive subdomains have not been adequately studied, including planning, decision-making, inhibition response, visual perception, and receptive language in particular. Notably, because the importance of social dysfunction after mTBI has been understated, additional research is required to improve understanding of how social impairments are related to brain structure and function. Future research should also aim to examine cognitive subdomains whose study has been relatively neglected, such as divided attention and ToM. Cognitive deficits should be examined as a function of injury severity because the lack of such stratification can result in inconclusive results which may conflict with those of other studies. Very importantly, future investigations should quantify the precise effects of age at injury and time since injury upon both post-traumatic cognition and neuroimaging correlates, given that such information was frequently found to be lacking from many studies. Comparison of mTBI patients as a function of their subsequent cognitive and clinical outcome would be particularly beneficial, since not all mTBI individuals continue to exhibit PCSs, and this effect may confound results if not taken into account.

Having systematically reviewed mTBI-related deficits and their cognitive assessment, our conclusion is that further knowledge synthesis in this research area requires future studies to focus on the rigorous and methodical assessment of cognitive subdomains and of their components, rather than on overarching cognitive domains, as still frequently done for attention, L&M and EF. Nevertheless, when evaluating cognitive function across entire cognitive domains, researchers should define and conceptualize cognitive function within such domains both thoroughly and systematically. The TBI research community should aim to clarify and establish consensus as to which specific deficits can be measured by commonly used cognitive assessments, and the relation of deficits within a certain domain to those within other domains and subdomains should be established. Because the accuracy of current classification schemes for cognitive categories, including that of the DSM-5, continues to be the subject of intense debate, TBI neuropsychologists and psychometricians should leverage their expertise and insights to assist the development and establishment of any novel taxonomies and hierarchies of cognitive functions. Additionally, factor analysis and similar methods should be used to clarify the relationships between commonly used assessment instruments and the cognitive categories advanced by the DSM-5 and by other taxonomies of cognition. Finally, novel assessments should be developed to assess cognition with high ecological validity.

The present review is not free of limitations. Many important factors relating to TBI patients’ performance on cognitive tests are beyond our scope; for example, we have only focused on adult TBI because pediatric TBI patients often exhibit patterns of impairment which are different from those observed in adults. Furthermore, we have not explored heterogeneities of impairment due to distinct injury mechanisms and clinical presentations. Distinctions between (A) complicated and uncomplicated mTBI, between (B) neuroimaging-free vs. CT- and MRI-informed conclusions and between (C) single vs. multiple injuries were not explored due to the scarcity of psychometric studies which account for these distinctions. Depending on the published research available, reviewed studies included both civilian and non-civilian participants; because these groups can inherently differ in several ways, knowledge synthesis across these two groups can be challenging and was not attempted.

## Author Contributions

MC and AI evaluated published literature, wrote the review and contributed to all drafting, editing, and revising. Both authors approved the final version of the submission and agreed to be accountable for the content of the work.

## Conflict of Interest

The authors declare that the research was conducted in the absence of any commercial or financial relationships that could be construed as a potential conflict of interest.
