# Supplementary material for: Neuroimaging and Psychometric Assessment of Mild Cognitive Impairment After Traumatic Brain Injury
Source: Front Psychol. 2020 Jul 7;11:1423. doi: 10.3389/fpsyg.2020.01423 (PMC7358255; doi:10.3389/fpsyg.2020.01423)
Supplement: Supplementary file 1 [file Table_1.DOCX]

|  | **Attention** | Sustained attention | Divided attention | Selective attention | Processing speed | **Learning & memory** | Free recall | Cued recall | Recognition memory | Episodic memory | Semantic memory | Implicit learning | **Executive function** | Planning | Decision-making | Feedback response | Working memory | Response inhibition | Cognitive flexibility | **Language** | Word-finding (naming) | Verbal fluency | Grammar and syntax | Receptive language | **Perceptual-motor function** | Visual perception | Visuo-construction | Perceptual-motor coordination |
| --- | --- | --- | --- | --- | --- | --- | --- | --- | --- | --- | --- | --- | --- | --- | --- | --- | --- | --- | --- | --- | --- | --- | --- | --- | --- | --- | --- | --- |
| SDMT | ✓ |  | ✓ |  | ✓ |  |  |  |  |  |  |  |  |  |  |  |  |  |  |  |  |  |  |  |  |  |  |  |
| DS | ✓ |  |  |  | ✓ |  |  |  |  |  |  |  |  |  |  |  | ✓ |  |  | ✓ |  |  |  |  |  |  |  |  |
| DSCT | ✓ |  |  |  | ✓ |  |  |  |  |  |  |  |  |  |  |  |  |  |  |  |  |  |  |  |  |  |  |  |
| TMT-A | ✓ |  |  |  | ✓ |  |  |  |  |  |  |  |  |  |  |  |  |  | ✓ |  |  |  |  |  |  |  |  |  |
| TMT-B | ✓ |  |  |  | ✓ |  |  |  |  |  |  |  | ✓ |  |  |  |  |  | ✓ |  |  |  |  |  |  |  |  |  |
| TAP | ✓ |  | ✓ |  |  |  |  |  |  |  |  |  |  |  |  |  |  |  |  |  |  |  |  |  |  |  |  |  |
| CPT | ✓ |  |  | ✓ |  |  |  |  |  |  |  |  |  |  |  |  |  | ✓ |  |  |  |  |  |  |  |  |  |  |
| ANT | ✓ |  |  | ✓ |  |  |  |  |  |  |  |  |  |  |  |  |  |  |  |  |  |  |  |  |  |  |  |  |
| SART | ✓ | ✓ |  |  |  |  |  |  |  |  |  |  | ✓ |  |  |  |  | ✓ |  |  |  |  |  |  |  |  |  |  |
| PVSAT | ✓ |  |  |  | ✓ |  |  |  |  |  |  |  |  |  |  |  |  |  |  |  |  |  |  |  |  |  |  |  |
| PASAT | ✓ | ✓ |  |  | ✓ |  |  |  |  |  |  |  |  |  |  |  | ✓ |  |  |  |  |  |  |  |  |  |  |  |
| MCT |  | ✓ |  |  |  |  |  |  |  |  |  |  |  |  |  |  |  |  |  |  |  |  |  |  |  |  |  |  |
| CRTT |  | ✓ |  |  | ✓ |  |  |  |  |  |  |  |  |  |  |  |  |  |  |  |  |  |  |  |  |  |  |  |
| DVT |  | ✓ |  |  |  |  |  |  |  |  |  |  |  |  |  |  |  |  |  |  |  |  |  |  |  |  |  |  |
| DTT |  |  | ✓ |  |  |  |  |  |  |  |  |  |  |  |  |  |  |  |  |  |  |  |  |  |  |  |  |  |
| CMT |  |  | ✓ |  | ✓ |  |  |  |  |  |  |  |  |  |  |  |  |  |  |  |  |  |  |  |  |  |  |  |
| SCWT |  |  |  |  | ✓ |  |  |  |  |  |  |  |  |  |  |  |  | ✓ | ✓ |  |  |  |  |  |  |  |  |  |
| VFT |  |  |  |  |  |  |  |  |  |  | ✓ |  | ✓ |  |  |  |  |  |  | ✓ |  | ✓ |  |  |  |  |  |  |
| NSTT |  |  |  | ✓ |  |  |  |  |  |  |  |  |  |  |  |  |  |  |  |  |  |  |  |  |  |  |  |  |
| CSAT |  |  |  | ✓ |  |  |  |  |  |  |  |  |  |  |  |  |  |  |  |  |  |  |  |  |  |  |  |  |
| RAVLT |  |  |  |  |  | ✓ | ✓ |  | ✓ |  |  |  |  |  |  |  |  |  |  |  |  |  |  |  |  |  |  |  |
| CVLT-II |  |  |  |  |  | ✓ | ✓ | ✓ | ✓ |  |  |  |  |  |  |  |  |  |  |  |  |  |  |  |  |  |  |  |
| MC 1 & 2 |  |  |  |  |  | ✓ |  |  |  |  |  |  |  |  |  |  |  |  |  |  |  |  |  |  |  |  |  |  |
| WMT |  |  |  |  |  | ✓ | ✓ | ✓ | ✓ |  |  |  |  |  |  |  |  |  |  |  |  |  |  |  |  |  |  |  |
| DPT |  |  |  |  |  | ✓ |  |  |  |  |  |  |  |  |  |  |  |  |  |  |  |  |  |  |  |  |  |  |
| WMS |  |  |  |  |  | ✓ |  |  |  | ✓ |  |  |  |  |  |  |  |  |  |  |  |  |  |  |  |  |  |  |
| IED |  |  |  |  |  | ✓ |  |  |  |  |  |  |  |  |  |  |  |  | ✓ |  |  |  |  |  |  |  |  |  |
| RCFT |  |  |  |  |  | ✓ |  |  |  |  |  |  |  |  |  |  |  |  |  |  |  |  |  |  |  |  | ✓ |  |
| FWM |  |  |  |  |  | ✓ |  |  |  |  |  |  |  |  |  |  |  |  |  |  |  |  |  |  |  |  |  |  |
| BSRT |  |  |  |  |  |  | ✓ |  |  |  |  |  |  |  |  |  |  |  |  |  |  |  |  |  |  |  |  |  |
| PN & SM |  |  |  |  |  |  |  |  |  |  | ✓ |  |  |  |  |  |  |  |  |  |  |  |  |  |  |  |  |  |
| ITTI |  |  |  |  |  |  |  |  |  |  |  | ✓ |  |  |  |  |  |  |  |  |  |  |  |  |  |  |  |  |
| SRTT |  |  |  |  |  |  |  |  |  |  |  | ✓ |  |  |  |  |  |  |  |  |  |  |  |  |  |  |  |  |
| TOHT |  |  |  |  |  |  |  |  |  |  |  | ✓ |  |  |  |  |  |  |  |  |  |  |  |  |  |  |  |  |
| HSCT |  |  |  |  |  |  |  |  |  |  |  |  | ✓ |  |  |  |  |  |  |  |  |  |  |  |  |  |  |  |
| BSAT |  |  |  |  |  |  |  |  |  |  |  |  | ✓ |  |  |  |  |  |  |  |  |  |  |  |  |  |  |  |
| COWAT |  |  |  |  |  |  |  |  |  |  |  |  | ✓ |  |  |  |  |  | ✓ |  |  | ✓ |  |  |  |  |  |  |
| PMT |  |  |  |  |  |  |  |  |  |  |  |  | ✓ |  |  |  |  |  |  |  |  |  |  |  |  |  |  |  |
| TOL |  |  |  |  |  |  |  |  |  |  |  |  | ✓ | ✓ |  |  |  |  |  |  |  |  |  |  |  |  |  |  |
| WCST |  |  |  |  |  |  |  |  |  |  |  |  | ✓ |  |  | ✓ |  |  | ✓ | ✓ |  |  |  |  |  |  |  |  |
| DKEFS |  |  |  |  |  |  |  |  |  |  |  |  | ✓ |  |  |  |  |  |  |  |  |  |  |  |  |  |  |  |
| NAB |  |  |  |  |  |  |  |  |  |  |  |  | ✓ |  |  |  |  |  |  |  |  |  |  |  |  |  |  |  |
| BADS |  |  |  |  |  |  |  |  |  |  |  |  |  | ✓ |  |  |  |  |  |  |  |  |  |  |  |  |  |  |
| PF A & B |  |  |  |  |  |  |  |  |  |  |  |  |  | ✓ |  |  |  |  |  |  |  |  |  |  |  |  |  |  |
| IGT |  |  |  |  |  |  |  |  |  |  |  |  |  |  | ✓ |  |  |  |  |  |  |  |  |  |  |  |  |  |
| N-Back |  |  |  |  |  |  |  |  |  |  |  |  |  |  |  |  | ✓ |  |  |  |  |  |  |  |  |  |  |  |
| G/N & SST |  |  |  |  |  |  |  |  |  |  |  |  |  |  |  |  |  | ✓ |  |  |  |  |  |  |  |  |  |  |
| LGST |  |  |  |  |  |  |  |  |  |  |  |  |  |  |  |  |  |  | ✓ |  |  |  |  |  |  |  |  |  |
| SP |  |  |  |  |  |  |  |  |  |  |  |  |  |  |  |  | ✓ |  |  |  |  |  |  |  |  |  |  |  |
| RIDR |  |  |  |  |  |  |  |  |  |  |  |  |  |  |  |  |  |  |  | ✓ |  |  |  |  |  |  |  |  |
| NST |  |  |  |  |  |  |  |  |  |  |  |  |  |  |  |  |  |  |  | ✓ |  |  | ✓ |  |  |  |  |  |
| BNT |  |  |  |  |  |  |  |  |  |  |  |  |  |  |  |  |  |  |  |  | ✓ |  |  |  |  |  |  |  |
| AWF |  |  |  |  |  |  |  |  |  |  |  |  |  |  |  |  |  |  |  |  | ✓ |  |  |  |  |  |  |  |
| TWFD |  |  |  |  |  |  |  |  |  |  |  |  |  |  |  |  |  |  |  |  | ✓ |  |  |  |  |  |  |  |
| RFFT |  |  |  |  |  |  |  |  |  |  |  |  |  |  |  |  |  |  |  |  |  | ✓ |  |  |  |  |  |  |
| AAT |  |  |  |  |  |  |  |  |  |  |  |  |  |  |  |  |  |  |  |  |  |  | ✓ |  |  |  |  |  |
| PPVT |  |  |  |  |  |  |  |  |  |  |  |  |  |  |  |  |  |  |  |  |  |  |  | ✓ |  |  |  |  |
| TT |  |  |  |  |  |  |  |  |  |  |  |  |  |  |  |  |  |  |  |  |  |  |  | ✓ |  |  |  |  |
| ACT |  |  |  |  |  |  |  |  |  |  |  |  |  |  |  |  |  |  |  |  |  |  |  | ✓ |  |  |  |  |
| CP 1 |  |  |  |  |  |  |  |  |  |  |  |  |  |  |  |  |  |  |  |  |  |  |  |  |  | ✓ |  |  |
| BDT |  |  |  |  |  |  |  |  |  |  |  |  |  |  |  |  |  |  |  |  |  |  |  |  |  |  | ✓ |  |
| LOJT |  |  |  |  |  |  |  |  |  |  |  |  |  |  |  |  |  |  |  |  |  |  |  |  |  |  | ✓ |  |
| HVOT |  |  |  |  |  |  |  |  |  |  |  |  |  |  |  |  |  |  |  |  |  |  |  |  |  |  | ✓ |  |
| BVRT |  |  |  |  |  |  |  |  |  |  |  |  |  |  |  |  |  |  |  |  |  |  |  |  |  |  | ✓ |  |
| CP 2 |  |  |  |  |  |  |  |  |  |  |  |  |  |  |  |  |  |  |  |  |  |  |  |  |  |  |  | ✓ |
| CP 3 |  |  |  |  |  |  |  |  |  |  |  |  |  |  |  |  |  |  |  |  |  |  |  |  |  |  |  | ✓ |

**Supplementary Table**. Psychometric instruments for the assessment of each cognitive domain (bold), including cognitive subdomains (cursive).
